# Super‐Resolution Vibrational Imaging Using Expansion Stimulated Raman Scattering Microscopy

**DOI:** 10.1002/advs.202200315

**Published:** 2022-05-06

**Authors:** Lixue Shi, Aleksandra Klimas, Brendan Gallagher, Zhangyu Cheng, Feifei Fu, Piyumi Wijesekara, Yupeng Miao, Xi Ren, Yongxin Zhao, Wei Min

**Affiliations:** ^1^ Department of Chemistry Columbia University New York NY 10027 USA; ^2^ Department of Biological Sciences Carnegie Mellon University Pittsburgh PA 15143 USA; ^3^ Department of Biomedical Engineering Carnegie Mellon University Pittsburgh PA 15143 USA; ^4^ Kavli Institute for Brain Science Columbia University New York NY 10027 USA

**Keywords:** chemical imaging, expansion microscopy, highly multiplexed nanoscale imaging, metabolic imaging, Raman nanoscopy

## Abstract

Stimulated Raman scattering (SRS) microscopy is an emerging technology that provides high chemical specificity for endogenous biomolecules and can circumvent common constraints of fluorescence microscopy including limited capabilities to probe small biomolecules and difficulty resolving many colors simultaneously. However, the resolution of SRS microscopy remains governed by the diffraction limit. To overcome this, a new technique called molecule anchorable gel‐enabled nanoscale Imaging of Fluorescence and stimulated Raman scattering microscopy (MAGNIFIERS) that integrates SRS microscopy with expansion microscopy (ExM) is described. MAGNIFIERS offers chemical‐specific nanoscale imaging with sub‐50 nm resolution and has scalable multiplexity when combined with multiplex Raman probes and fluorescent labels. MAGNIFIERS is used to visualize nanoscale features in a label‐free manner with C—H vibration of proteins, lipids, and DNA in a broad range of biological specimens, from mouse brain, liver, and kidney to human lung organoid. In addition, MAGNIFIERS is applied to track nanoscale features of protein synthesis in protein aggregates using metabolic labeling of small metabolites. Finally, MAGNIFIERS is used to demonstrate 8‐color nanoscale imaging in an expanded mouse brain section. Overall, MAGNIFIERS is a valuable platform for super‐resolution label‐free chemical imaging, high‐resolution metabolic imaging, and highly multiplexed nanoscale imaging, thus bringing SRS to nanoscopy.

## Graphical abstract

This work develops a new technique called MAGNIFIERS that integrates stimulated Raman scattering (SRS) microscopy with expansion microscopy for super‐resolution vibrational imaging. MAGNIFIERS revitalizes a full spectrum of utilities of SRS imaging into the sub‐diffraction regime with an effective resolution below 50 nm and general sample compatibility. Specifically, it supports super‐resolution label‐free chemical imaging, high‐resolution metabolic imaging, and highly multiplexed nanoscale imaging.

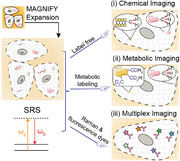

## 1 Introduction

While fluorescence microscopy has been the predominant imaging modality in biophotonics, it faces fundamental limitations for many advanced imaging applications. Going beyond fluorescence, stimulated Raman scattering (SRS) microscopy is emerging as a powerful modality for illuminating otherwise invisible chemical bonds of biomolecules in complex biological contexts.^[^ ^1^, ^2^ ^]^ In contrast to fluorescence imaging, SRS generates high chemical specificity via probing vibrational transitions, enabling label‐free detection of vital biomolecules of proteins, lipids, nucleic acids, etc.^[^ ^1^, ^3^ ^]^ Moreover, integration of SRS with vibrational tags renders a paradigm of bioorthogonal chemical imaging for studying metabolic activities in cells and tissues.^[^ ^2^, ^4^, ^5^ ^]^ The tiny size and good biocompatibility of vibrational tags, such as stable isotopes and triple bonds, enable nonperturbative tracing of small molecules,^[^ ^4^ ^]^ which is difficult to achieve with fluorescence because of the bulky fluorophores. Furthermore, by virtue of much narrower vibrational peaks (≈10 cm^–1^) compared to fluorescence peaks (≈500 cm^–1^), Raman imaging offers scalable multiplexity and breaks the “palette number barrier” of fluorescence, which can typically only image 4–5 colors simultaneously due to the overlap of inherently broad fluorescence spectra. In this regard, development of rainbow‐like Raman dyes leverages the sensitivity of SRS and opens the door for super‐multiplexed vibrational imaging.^[^ ^6^, ^7^, ^8^ ^]^ In particular, by combining electronic preresonance spectroscopy with stimulated Raman scattering (SRS) microscopy (i.e., epr‐SRS), the Raman cross sections of electronically coupled vibrational modes in light‐absorbing dyes can be enhanced by 10^13^‐fold.^[^ ^6^, ^9^ ^]^ Owing to this drastic enhancement, we achieved nanomolar sensitivity of Raman‐active dyes (such as commercial far‐red fluorescent dyes and specially‐designed manhattan raman scattering (MARS) probes), and demonstrated epr‐SRS imaging of specific proteins.^[^ ^6^, ^10^, ^11^ ^]^

Collectively, SRS imaging circumvents the fundamental bottlenecks of fluorescence microscopy and provides unique benefits including label‐free chemical imaging of endogenous biomolecules,^[^ ^1^, ^12^ ^]^ nonperturbative mapping of small metabolites,^[^ ^4^ ^]^ and simultaneous profiling of a large group of specific biomarkers.^[^ ^6^, ^11^ ^]^ Yet, being an optical technique that uses near‐infrared or visible light, SRS microscopy is still constrained to diffraction‐limited spatial resolution (≈300 nm). Thus, many crucial nanoscale organizations in cells and tissues cannot be resolved by the current iteration of SRS microscopy. Despite extensive efforts in extending Raman imaging beyond the diffraction limit, sophisticated instrumentation is required and only moderate resolution improvement (typically less than twofold) has been achieved, resulting in limited utilization in biological specimens.^[^ ^13^, ^14^, ^15^, ^16^, ^17^, ^18^ ^]^ Hence, vibrational‐based nanoscale imaging approaches are still an underexplored territory in biomedical research.

Over the last half decade, a sample‐oriented nanoscale imaging technique known as expansion microscopy (ExM) has emerged as a nonoptical means of achieving effective resolutions beyond the diffraction limit. ExM is a powerful technique for nanoscale optical imaging through physical magnification of tissue samples embedded in a water‐swellable polymer gel.^[^ ^19^ ^]^ ExM was initially developed for fluorescence microscopy with an ≈4.5 × expansion in each dimension in pure water and quickly evolved to different variants with widespread use in biology and pathology.^[^ ^20^, ^21^, ^22^, ^23^, ^24^ ^]^ Conceptually, the principle of ExM could be applied to overcome the diffraction limit of SRS imaging. However, common ExM variants^[^ ^22^, ^23^, ^25^ ^]^ rely on strong protease digestion to facilitate expansion, thereby destroying endogenous proteins. On the other hand, ExM variants that retain proteins after expansion^[^ ^26^, ^27^, ^28^ ^]^ are more compatible with SRS imaging, but they require special fixation protocols and are only validated on culture cells and mouse brain tissues. A very recent technique called VISTA was developed for label‐free chemical imaging of proteins on expanded mouse brain tissues using a protein‐retention ExM variant,^[^ ^29^ ^]^ but it inherits the critical limitation of this ExM variant on tissue compatibility and resolution gain.

Recently, a new ExM framework has emerged that offers larger expansion factors along with broad biomolecule retention and tissue compatibility,^[^ ^30^ ^]^ capable of expanding a multitude of tissue types with standard fixation, from PFA‐fixed mouse brain and human lung organoid to an array of clinically relevant formalin‐fixed paraffin‐embedded (FFPE) human tissues that have proved challenging to expand in the past. By combining SRS imaging with this framework, we lay out an approach named Molecule Anchorable Gel‐enabled Nanoscale Imaging of Fluorescence and stimulated Raman Scattering microscopy (MAGNIFIERS). Harnessing the superior retention of biomolecular contents and general sample compatibility across multiple tissue types and fixation methods of the new ExM framework, the MAGNIFIERS approach provides excellent reversible linear expansion up to ≈7.2‐fold providing lateral resolutions of ≈41 nm (298 nm/7.2 = 41.4 nm) and axial resolutions of ≈194 nm (1400 nm/7.2 = 194 nm), with great flexibility to couple vibrational metabolic labeling and Raman dye staining.

**Figure 1:**
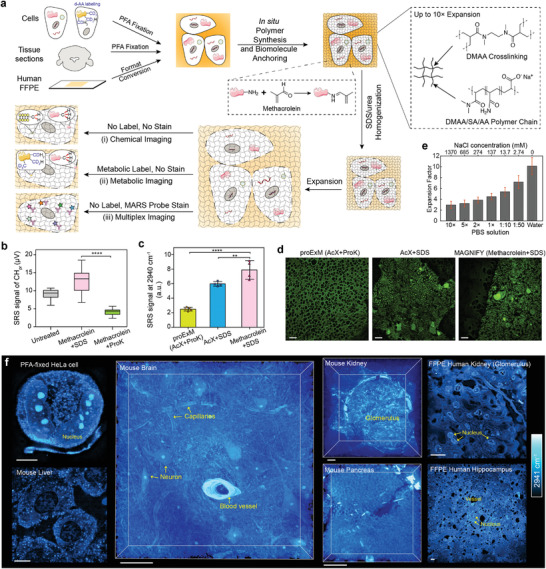
Integration of SRS with an optimized ExM protocol (MAGNIFY). a) Schematics of the workflow of MAGNIFIERS and gel chemistry of MAGNIFY. During in situ polymer synthesis, methacrolein was added as a part of the monomer solution to efficiently attach biomolecules to the growing matrix. The gel was formed from polymerization of cocktails of the monomers acrylamide (AA, nonionic monomer), sodium acrylate (SA, ionic monomer) and *N,N*‐dimethylacrylamide (DMAA, nonionic self‐cross‐linker. Afterward, the gel‐embedded specimens were treated with sodium dodecyl sulfate (SDS)/urea solution in 80–90 °C for at least 1 h for homogenization. PFA, paraformaldehyde. b) Quantification of protein contents for untreated and expanded (expanded with methacrolein‐linking and SDS/urea homogenization or Proteinase K digestion) mouse kidney. SRS signals were plotted as mean ±  s.d. (*n* = 29, 25, 25 regions of interest (ROIs)). Absolute signal in the untreated sample is converted with a volume dilution factor of 4.3^3^ ≅ 80‐fold after expansion. Two‐tailed unpaired *t*‐test, ****p < 0.0001, *t *= 13. c) Comparison of protein retention on the proExM protocol with the MAGNIFIERS protocol. SRS signals were plotted as mean ±  s.d. (*n* = 5, 4, 4 ROIs). One‐way ANOVA followed by Bonferroni's post hoc test using the “Methacrolein+SDS” as the control column, ****p < 0.0001 and **p = 0.0085. d) SRS images of CH_3_ at 2941 cm^–1^ in the mouse brain tissues. (left) expanded in proExM protocol with AcX as the crosslinker and Proteinase K digestion, (middle) expanded with AcX as the crosslinker and SDS/urea homogenization, (right) expanded with methacrolein‐linking and SDS/urea homogenization. e) Measurement of expansion factors (mean ± s.d.) in PBS buffers with different salt concentrations. Corresponding salt concentrations of used PBS buffer solution are labeled on the up *x*‐axis. Values of *n* are provided in Table S2 (Supporting Information). f) 3D‐rendered SRS images of CH_3_ peak at 2941 cm^–1^ of PFA‐fixed HeLa cell, mouse brain, liver, kidney, and pancreas tissues, and FFPE human kidney and brain hippocampus tissue. Expansion factors are 4.5 for HeLa, organoid and mouse brain, 4.3 for mouse kidney, liver, and pancreas, 3.8 for FFPE human kidney and FFPE human spleen, 2.0 for FFPE human hippocampus in 1× PBS. Scale bars, 10 µm in (d,f) and calibrated in biological scale.

MAGNIFIERS revitalizes a full spectrum of utilities of SRS imaging into the sub‐diffraction regime (**Figure** **1**a). Specifically, we show that MAGNIFIERS supports label‐free nanoscale imaging of endogenous biomolecules including proteins, lipids, and DNA. We further demonstrate its power for high‐resolution metabolic imaging of protein aggregates in cells labeled with deuterium‐substituted amino acids. Finally, we use MAGNIFIERS to map the nanoscale locations of eight different markers in mouse brain tissue in one shot by applying multiplex Raman labels and conventional fluorescent probes.

## 2 Results

### 2.1 Integration of SRS with an Optimized Biomolecule‐Retention ExM Protocol (MAGNIFY)

To fully demonstrate the potential of expansion SRS imaging, we chose a new ExM strategy (Figure 1a) that achieves a favorable balance between biomolecule retention, expansion factor, and sample generality.^[^ ^30^ ^]^ The new method is called Molecule Anchorable Gel‐enabled Nanoscale In situ Fluorescence MicroscopY (MAGNIFY).^[^ ^30^ ^]^ To demonstrate its utility for SRS imaging, we first quantified the protein retention rate by imaging CH_3_ stretching vibration of proteins at 2941 cm^–1^ in paraformaldehyde (PFA)‐fixed tissues pre‐ and post‐expansion (Figure 1b–d; Figure S1, Supporting Information). Remarkably, we found a near 100% protein retention rate using the MAGNIFY approach (Figure 1b), substantially higher than that of protein‐retention expansion microscopy using 6‐((acryloyl)amine) hexanoic acid (AcX) as an anchoring agent (i.e., proExM,^[^ ^22^ ^]^ Figure 1c,d), ensuring optimal sensitivity and effective resolution. The reasons for the optimized biomolecule retention rate are twofold. First, methacrolein was employed as part of the monomer solution to efficiently attach biomolecules to the growing matrix during the in situ polymerization step, eliminating the need for stepwise reactions of anchoring and polymerization (Figure 1a). Second, rather than strong protease digestion, the gel‐embedded specimens were treated with a denaturant‐rich solution at 80–90 °C for homogenization to ensure maximum retention of biomolecules in the gel.

MAGNIFIERS relied on a reformulated hydrogel (Figure 1a) that allows isotropic physical expansion up to tenfold in a single round.^[^ ^30^ ^]^ To theoretically estimate the improved spatial precision, we calibrated the resolution of the current SRS microscopy by imaging single 100‐nm polystyrene beads at 3056 cm^–1^ (Figure S2, Supporting Information). Cross‐section profiles fitted by the Gaussian function indicated the full width at half maximum (FWHM) of lateral direction of 298 nm (Figure S2a, Supporting Information). Thus, an effective resolution of ≈30 nm is possible given an expansion factor (EF) of ≈10 (Figure 1e). Unless stated otherwise, the reformulated hydrogel was used for expanding all biological specimens.

MAGNIFIERS is broadly applicable to a wide range of specimens (Figure S3, Supporting Information), as it is compatible with common fixation methods and multiple tissue types, including mechanically tough tissues such as kidney and liver. For instance, SRS imaging of CH_3_ stretching at 2941 cm^–1^ visualized rich 3D structural information in cultured cells and mouse tissues such as the brain, liver, kidney, and pancreas (Figure 1f; Video S1, Supporting Information), thanks to the intrinsic 3D sectioning capability from the two‐photon excitation nature of SRS. In addition, MAGNIFIERS is suitable for human organoids (Figure S3, Supporting Information), a favorable model system for human biology and medicine. Going beyond freshly preserved cells and tissues, archival samples such as FFPE sections of human brain, kidney, and spleen, which is a popular form of preparation for biopsy specimens that aids in clinical examination and pathology research, are also compatible with MAGNIFIERS (Figure 1f; Figure S3, Supporting Information).

### 2.2 Label‐Free Visualization of Biological and Pathological Ultrastructures

**Figure 2:**
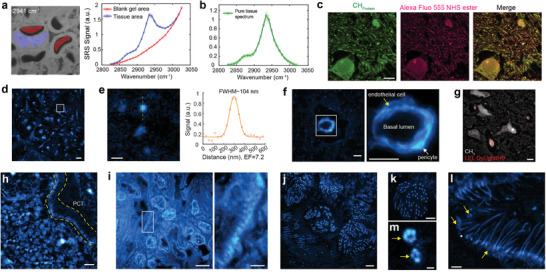
MAGNIFIERS visualizes ultrafine structures of biological samples via label‐free protein imaging. a) Hyperspectral SRS imaging of the C—H stretching region on expanded FFPE human kidney. Left, masks of tissue area (red) and blank gel area (blue) for spectral analysis labeled on the SRS image of 2941 cm^–1^. Right, SRS spectra of two masked areas. Single‐frequency SRS images are in Figure S4a (Supporting Information). b) Background‐removed SRS spectrum of expanded FFPE human kidney. c) SRS CH_3_ image at 2941 cm^–1^ and fluorescence image of Alexa Fluor 555 NHS ester labeled mouse brain tissue. d) CH_3_ image of extended mouse brain in 1/50 × PBS (7.2‐fold expansion) with a 1.2 NA objective. e) Zoom into the region outlined by the white box in (d). Right, line profile of a sub‐diffraction‐limited spot outlined by the yellow line. f) Fine structures of blood–brain barrier in mouse brain tissue. Single endothelial cells and a pericyte were visualized. Images were collected using a 1.05 NA objective in 1× PBS (4.5‐fold expansion). Right, zoom into the region outlined by the white box. g) Blood vessels and capillaries stand out in the protein channel, confirmed by lectin staining. Expanded mouse brain tissue labeled with *L. Esculentum* lectin (LEL)‐DyLight 649 and imaged with a 1.05 NA objective in 1× PBS (4.5‐fold expansion). h) SRS protein image in expanded mouse kidney. PCT, proximal convoluted tubule. Yellow region highlights the cilia on the surface of PCT. Images were collected using a 1.05 NA objective in 1× PBS (4.3‐fold expansion). i) SRS protein image reveals periodic podocyte foot processes in expanded FFPE human kidney tissue. Right, zoom into the region outlined by the white box. Images were collected using a 1.2 NA objective in 1× PBS (3.8‐fold expansion). j–m) Flower‐like cilia structure (j,k) and the ring structure at the base of cilia (l,m) in the expanded human lung organoid were visualized by the protein channel at 2941 cm^–1^. Yellow arrows in (k,m) highlight the basal bodies. Images were collected with a 1.05 NA objective, (j,l) in 1× PBS (4.5‐fold expansion) and (k,m) in 1/50 × PBS (7.2‐fold expansion). Scale bars (in biological scales), 200 nm in (e,m); 1 µm in (d,i (right), k,l); 2 µm in (f,h,j); 5 µm in (c,g,i (left)).

Encouraged by the near 100% protein retention, we envision that it is advantageous for unveiling nanoscale features without the need to label or stain with fluorescent dyes. In standard SRS microscopy, C—H stretching region at 2800–3050 cm^–1^ is desirable to image endogenous species.^[^ ^3^ ^]^ However, the expanded sample might exhibit O—H background (2900–3700 cm^–1^) from the water (typically >90% by volume) in the sample and C—H background from the gel matrix itself, thus potentially overwhelming the signal of interest. To study this, we performed hyperspectral SRS analysis of expanded FFPE human kidney tissues (**Figure** **2**a; Figure S4a, Supporting Information). We chose FFPE tissue as a test because of its simple Raman spectrum, as protein is the major component and most lipids are removed in deparaffinization before expansion (Figure S4b, Supporting Information). By analyzing the spectra of a tissue region and a blank gel region (Figure 2a, left), we observed strong C—H signals on the tissue area and the spectral shape in the blank gel region indicates the gel background is dominated by water O—H vibration (Figure 2a, right). After subtracting the water background, which is broad and smooth, the spectral shape recapitulates a typical CH_3_ mode of proteins with a major peak at ≈2941 cm^–1^ (Figure 2b), consistent with the fact that proteins are the major anchored biomolecules in FFPE tissue. We also observed the gel C—H background whose intensity depends on both gel recipe and embedded samples (Figure S4c, Supporting Information). By replacing the water inside the gel with heavy water, we confirmed the spectral shape of gel C—H signals and their uniform spatial patterns (Figure S4d,e, Supporting Information). Based on these spectral analyses, we concluded that the uniform gel backgrounds can be readily subtracted, validating using the CH_3_ vibration at 2941 cm^–1^ for imaging total proteins in expanded specimens.

It is valuable to compare the protein distribution pattern from SRS imaging of CH_3_ vibration at 2941 cm^–1^ to that from fluorescence imaging of stained total protein content.^[^ ^31^, ^32^ ^]^ By performing SRS and fluorescence imaging on an expanded mouse brain section stained with *N*‐hydroxysuccidimidyl (NHS) ester‐dye conjugates, we observed similar patterns (Figure 2c). This morphological agreement between SRS and fluorescence images corroborates our prior spectral analysis.

The effective resolution is jointly determined by the objective's numerical aperture (NA) and the achievable EF, which can be controlled by osmotic pressure of the imaging solution (Figure 1e). Given a practical EF of 7.2 with good signal (Figure 2d), an effective resolution of ≈298/7.2 = 41 nm is feasible. With this improved resolution, we resolved sub‐diffraction‐limited dots on expanded mouse brain tissue, proved by a fitted FWHM of 104 nm (Figure 2e).

MAGNIFIERS can unveil a broad range of biological or pathological ultrastructure features solely via label‐free protein imaging. First, the microscopic view of blood–brain barrier in mouse brain tissue, including single endothelial cells and a pericyte, was visualized (Figure 2f), supported by the co‐staining with lectins as a blood vessel marker (Figure 2g; Figure S4f, Supporting Information). Benefiting from the good sample compatibility of MAGNIFY, we further revealed interesting nanoscale architectures in both PFA‐fixed mouse kidney and FFPE human kidney specimens. For instance, cilia were visualized at the inner layer of the proximal convoluted tubule (PCT) in mouse kidney (Figure 2h). In addition, we observed sub‐diffraction‐limited structures of interdigitated foot processes of podocytes in the glomeruli of human (Figure 2i) and mouse kidney tissues (Figure S5a–e, Supporting Information), which is a clinical metric for many nephrotic kidney diseases such as kidney minimal change disease.^[^ ^33^ ^]^ Moreover, in expanded human bronchial epithelial cells‐derived lung organoids, flower‐like cilia architectures were faithfully elucidated, projecting from the apical epithelial surface (Figure 2j,k; Figure S5f and Video S2, Supporting Information). Intracellular structures and patterns of surrounding apoptotic bodies were also clearly observed (Figure S5g,h, Supporting Information). Remarkably, at the basal foot of cilia, the ring structure of ciliary base bodies, which are composed of a 9 + 0 microtubule arrangement and have an inner diameter of ≈200 nm,^[^ ^34^ ^]^ were precisely identified (Figure 2l,m, yellow arrows).

### 2.3 Label‐Free Nanoscale Imaging of Chemical Compositions

**Figure 3:**
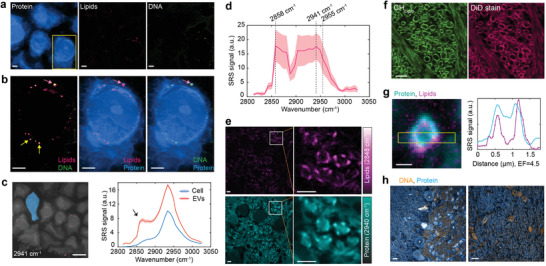
Label‐free nanoscale imaging of chemical compositions. a) Spectrally unmixed C—H channels for protein, lipids, and DNA in expanded human lung organoid. b) Two‐color overlayed images of zoom‐in areas outlined by the yellow box in (a). Arrows indicate EVs containing lipids and DNA. Images were collected using a 1.05 NA objective in 1× PBS (4.5‐fold expansion). c) SRS spectral analysis of the cell area (blue) and extracellular vesicles (EVs, red) in the human lung organoid. Left, selected areas for spectral analysis representing the cell (blue) and EVs (red). Right, background‐subtracted SRS spectra. Arrow indicated a side peak ≈2865 cm^–1^ contributing from the CH_2_ signal of lipids in small EVs. Raw hyperspectral SRS spectra and all single‐frequency SRS images are in Figure S6a (Supporting Information). d) Hyperspectral SRS spectrum at C—H region of a single EV in (b) marked by a pink arrow. e) Spectrally unmixed C—H channels for protein and lipids in the expanded mouse brain tissue. Right, zoom into areas outlined by the white boxes. Images were collected using a 1.05 NA objective in 1× PBS (2.0‐fold expansion, original ExM gel). f) CH_Lipid_ and DiD stain images of expanded mouse cerebellum. g) Transverse cross‐section of an individual axon in the expanded mouse brain tissue. Images were collected using a 1.05 NA objective in 1/25 × PBS (2.6‐fold expansion, original ExM gel). Right, corresponding plot of the intensity of proteins and lipids along the yellow box long axis. h) Overlayed images of DNA and protein in the expanded mouse cerebellum. Shaded areas in (c,d) indicate the s.e.m. from different pixels. Scale bars (in biological scales), 500 nm in (g); 2 µm in (a,b,e); 5 µm in (f); 10 µm in (c,h).

The MAGNIFY framework is known to retain biomolecules other than proteins such as lipids and nucleic acids (in Supporting Information).^[^ ^30^ ^]^ Thus, MAGNIFIERS holds great promise for label‐free chemically‐defined nanoscale imaging in a comprehensive view. Yet, the uncharacterized issue is whether SRS can detect these molecules with sufficient contrast. Prior SRS spectral studies have dissected the C—H signals into contributions from proteins (≈2931 cm^–1^), lipids (≈2854 cm^–1^), and DNA (≈2956 cm^–1^) through linear spectral unmixing.^[^ ^12^ ^]^ We started by applying this strategy to analyze expanded human lung organoids with an EF of 4.5. In the unmixed images that are labeled as CH_Pr_, CH_L_, and CH_DNA_ (**Figure** **3**a,b), we observed the presence of lipids in small‐size extracellular vesicles^[^ ^35^ ^]^ (EVs, secreted membrane vesicles), some of which also contain CH_DNA_ signals. We next performed a hyperspectral analysis of the entire C—H region (Figure S6a, Supporting Information). By examining the pixel‐averaged SRS spectrum of all small EVs (i.e., exosomes) in the region of interest, a distinct side peak ≈2865 cm^–1^ appeared (Figure 3c; Figure S6b, Supporting Information), in agreement with the peak signature of CH_2_ vibration from lipids. Exosomes contain high levels of cholesterol, sphingomyelin, and ceramide, similar to the composition of detergent‐resistant lipid rafts.^[^ ^36^ ^]^ As a control, the SRS spectrum of the cell predominantly reflects the chemical footprint of proteins (Figure 3c, blue). Furthermore, we generated the Raman spectrum of a single DNA‐containing EV (pink arrowed in Figure 3b) that displayed another pronounced shoulder peak of ≈2955 cm^–1^ originating from DNA as we anticipated (Figure 3d; Figure S6c, Supporting Information). This observation is in line with prior reports demonstrating that EVs contain proteins, lipids, and nucleic acids.^[^ ^35^, ^37^ ^]^

EVs have been growingly recognized to play crucial roles in diverse biological systems to shuttle molecules across cells, showing promise to aid in disease diagnosis or to be engineered to deliver therapeutic payloads.^[^ ^38^ ^]^ However, imaging EVs at the single‐vesicle level remains a challenging task due to their small size and potential labeling bias.^[^ ^39^ ^]^ Here, we have demonstrated that MAGNIFIERS enables analyzing chemical compositions of single exosomes in their spatial context without labeling (Figure 3a–d). It is noteworthy that we only observed lipids in small EVs at submicron size, but not others such as large microvesicles and apoptotic bodies (Figure S6d, Supporting Information). This could be explained by the differential lipid compositions of EVs determined by their biogenesis processes.^[^ ^35^ ^]^ In this regard, the existence of CH_L_ signals may work as a marker to identify EV biogenesis.

We also observed interesting patterns on the CH_L_ channel of the expanded mouse brain under expansion levels of up to 4.5‐fold (Figure 3e; Figure S7, Supporting Information). Despite the relatively harsh homogenization treatment with detergents and heating, we still found that ≈43% lipids have been retained for the mouse brain (Figure S8, Supporting Information). Additionally, the fluorescence stain of lipids with hydrophobic dye DiD colocalized with CH_L_ channel very well (Figure 3f; Figure S9, Supporting Information). Transverse cross‐section of myelinated axons was clearly unveiled in the CH_L_ channel across the brain tissue (Figure 3e,f). Axon fibers are enwrapped by myelin, a multilayered membranous sheath that serves as an electrical insulator for the axon to provide rapid conduction of action potentials. Myelin contains a high content of lipids made of large amounts of cholesterol and phospholipids (e.g., sphingomyelin), a very similar composition to the exosomes studied above. Zooming into an individual myelinated axon, the lipids signal appeared as a hollow structure with a core axon and a myelin covering (Figure 3h). Besides lipids, DNA patterns were clearly visualized in the expanded mouse brain via the CH_DNA_ channel (Figure 3i).

Overall, MAGNIFIERS cannot only examine the ultrafine structures but also advance in revealing their chemical compositions, such as lipids and nucleic acids.

### 2.4 High‐Resolution Metabolic Imaging of Huntingtin Aggregates

Metabolic activities are the outcome of gene expression and relate to many dysfunctions and diseases. Bioorthogonal chemical imaging is a new paradigm that utilizes “chemical bonds” as tiny vibrational tags for profiling small metabolites in cells and animals.^[^ ^40^, ^41^, ^42^ ^]^ Different from bulky fluorophores that easily affect the function of the attached small metabolites, vibrational tags are minimally perturbative to normal biological functions. Among them, carbon—deuterium bonds are particularly effective with little perturbation, broad accessibility, and residing in the bioorthogonal spectral window (where no endogenous biomolecules vibrate). Leveraging the power of small vibrational tags, MAGNIFIERS shall be able to gauge nanoscale features of metabolic activity.

**Figure 4:**
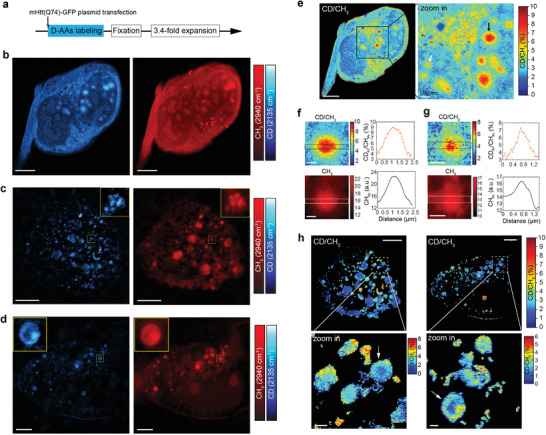
Super‐resolution metabolic imaging of newly synthesized protein in Huntingtin aggregates. a) Deuterated amino acids (D‐AAs) labeling in time with simultaneous expression of mutant huntingtin (mHtt74Q‐GFP) proteins for 48 h. The cartoons display the experimental pipeline of plasmid transfection, medium exchanges, fixation, and expansion. b–d) Representative ROIs of CH_3_ images at 2941 cm^–1^ and CD images at 2135 cm^–1^ after expansion. Up in (c,d), zoom into the region outlined by the yellow box. e) Ratio images of CD/CH_3_ for ROI in (b). Right, zoom into the region outlined by the black box. Arrow pointed out representative aggregates analyzed in (f,g). f) Analysis of the aggregate pointed with black arrow in (e). g) Analysis of the aggregate pointed with white arrow in (e). h) Ratio images of CD/CH_3_ for ROI in (c,d). Down, zoom into the regions outlined by the dotted box. All images were collected with a 1.05 NA objective in 5× PBS (3.44‐fold expansion). Scale bars (in biological scales), 5 µm in (b–d, e (left), h (up)); 500 nm in (f–g, e (right), h (down)).

Abnormal protein aggregation is associated with many neurodegenerative diseases. Aggregation‐prone polyglutamine (polyQ) protein is directly linked to Huntington's disease pathogenesis. Recent evidence suggests metabolic dysregulation in aggregates, which could be a potential target for future therapies.^[^ ^43^ ^]^ Being able to visualize fine structures of protein synthesis in small aggregates in the cellular environment could gain mechanistic insights and uncover therapeutic opportunities. While super‐resolution fluorescence imaging techniques have been extensively applied to study polyQ aggregates on resolving fine details of structures,^[^ ^44^, ^45^ ^]^ they have failed to provide information on metabolic activities of these aggregates. Using deuterium‐labeled amino acids (D‐AAs),^[^ ^41^ ^]^ here, we demonstrate the application of MAGNIFIERS for metabolic imaging by studying protein synthesis of polyQ. After concurrently expressing polyQ proteins and labeling newly synthesized proteins with D‐AAs in HeLa cells (Figure S10a, Supporting Information), we fixed the cells, then gelled and linearly expanded them ≈3.4‐fold in each dimension (**Figure** **4**a). Formation of PolyQ aggregates with different patterns (such as large inclusion bodies (IB s), fibrils, and branched clusters) were observed in both the cytosol and nucleus through imaging the CH_3_ peak at 2941 cm^–1^ or the CD peak at 2135 cm^–1^ (Figure 4b–d). The improved spatial resolution is beneficial for resolving closely packed small clusters (Figure 4c, inset) and the heterogeneous patterns of individual protein aggregates (Figure 4d, inset).

Interestingly, the spatial distributions of new proteins (CD channel) and old proteins (CH_3_ channel) were not always consistent. Quantitatively, the protein synthesis dynamics can be evaluated via the ratio of CD intensity over CH_3_ intensity (Figure 4e–i). In a cell with multiple IBs located in the nucleus, we observed higher CD/CH_3_ values in the core of both large (Figure 4f) and small (Figure 4g) IBs. This may suggest different phases of aggregates formation: the initial seeding of aggregates rely on active loading of misfolded polyQ and later aggregates expansion recruit not only misfolded polyQ but more other surrounding normal proteins. However, in cells with abundant aggregate formation and severely impaired cellular structures, we observed higher metabolic activities close to the boundary of some of our aggregates (Figure 4h), which may be generated through the fragmentation of large aggregates. In addition, through labeling different time periods of polyQ expression, we can monitor the growth trajectory of aggregates. For example, with pulse‐chase labeling using regular medium first and D‐AAs replaced medium later, we found large clusters of aggregates are metabolically inactive in a long time period (Figure S10b, Supporting Information).

Overall, MAGNIFIERS platform can map metabolic activities of protein synthesis and turnover with a sub‐diffraction‐limited spatial resolution of ≈100 nm. We demonstrated its utility in the context of studying polyQ aggregates inside mammalian cells, which shows various nanoscale patterns of new protein synthesis. The observed features (core and boundary) of polyQ aggregates all have sub‐diffraction‐limited length scales. While we demonstrated with D‐AAs here for studying protein synthesis, this principle can be applied to many other cases such as tracing lipid uptake with deuterium‐labeled fatty acids and detecting DNA synthesis with 5‐ethynyl‐2′‐deoxyuridine (EdU).

### 2.5 Nanoscale Raman Dye Imaging

Multiplexed super‐resolution protein imaging in 3D tissues remains a technical challenge. Limited by the broad excitation and emission bandwidth of fluorescence, it is fundamentally difficult for regular super‐resolution techniques to detect more than four targets. While researchers have demonstrated super‐resolution imaging of multiple targets using direct STORM,^[^ ^46^ ^]^ gel embedding and expansion,^[^ ^26^, ^47^ ^]^ and DNA‐PAINT,^[^ ^48^, ^49^ ^]^ they all relied on several rounds of staining, imaging, stripping and/or restaining. These cyclic methods could be artifact‐prone, considering fine structures are vulnerable to nonlinear histological changes among cycles and necessitate higher precision on image alignment. To overcome these technical difficulties, we plan to employ a one‐shot multiplexing approach, by leveraging much narrower vibrational transitions.

Recently, we developed a novel multiplexing method of electronic pre‐resonant SRS (epr‐SRS) that detects electronically coupled vibrational modes in far‐red absorbing dyes with sub‐micromolar sensitivity. Probe‐wise, a specially engineered MARS dye palette with *π*‐conjugated triple bonds has been developed, which generates single and sharp peaks at the cell‐silent region.^[^ ^6^, ^8^ ^]^ Epr‐SRS enables Raman detection of interesting biomarkers ranging from proteins, transcripts, and enzyme activities.^[^ ^6^, ^50^ ^]^ In particular, immuno‐eprSRS with MARS dye‐conjugated antibodies has emerged as an encouraging platform for one‐shot multiplexed protein imaging,^[^ ^6^, ^11^ ^]^ bypassing technical troubles involved in cyclic techniques. Yet, it only works in a diffraction‐limited paradigm.

**Figure 5:**
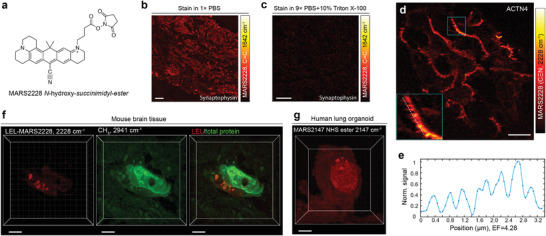
Nanoscale Raman dye imaging. a) Chemical structure of NHS ester functionalized MARS2228. b) Staining background when 1× PBS was used as the staining buffer. c) Correct staining patterns of Synaptophysin using 9× PBS with 10% Triton‐X as the staining buffer. d) Immuno‐eprSRS image of actinin‐4 (ACTN4, specifically label tertiary podocyte foot processes) in FFPE human kidney tissue with MARS2228. Inset, zoom into the region outlined by the blue box, dotted white curve within the inset indicates the line cut analyzed below. e) Normalized epr‐SRS signal of the nitrile mode of MARS2228 along the line cut of the inset in (d). f) 3D‐rendered epr‐SRS image of MARS2228‐conjugated *L. Esculentum* lectin (LEL) labeling and CH_3_ image of 2941 cm^–1^. g) 3D‐rendered epr‐SRS image of expanded human lung organoid stained with MARS2147 NHS ester dye. Images were collected using a 1.05 NA objective (d) in 1/50 × PBS (4.28‐fold expansion, human kidney ExPath gel), (b,c, f–g) in 1× PBS (4.5‐fold expansion, mouse brain MAGNIFY gel). Scale bars (in biological scales), 10 µm in (b,c); 5 µm in (d,f,g).

We first studied the interplay between epr‐SRS and MAGNIFY. Because the enzyme‐free homogenization step enables post‐expansion labeling (Figure 1a), more epitopes may be revealed,^[^ ^51^ ^]^ thus enhancing the labeling efficiency and overall signal size of immuno‐eprSRS. We tested immuno‐eprSRS in MAGNIFIERS using MARS2228 NHS ester conjugated secondary antibody (dye structure in **Figure** **5**a) to stain the expanded mouse brain section. However, we observed nonspecific staining backgrounds to varying degrees when the regular staining buffer of 1× PBS was utilized (Figure 5b; Figure S11a,b, Supporting Information). Considering MARS dyes are positively charged and the gel matrix contains negatively charged carboxyl groups, we reasoned this nonspecific staining might originate from an electrostatic effect. Hence, we changed the staining and washing buffers to a high‐salt solution of 9× PBS (charge shielding effect) and supplemented with high‐concentration Triton X‐100 (10%) that has been reported to prompt antibody binding.^[^ ^52^ ^]^ Effectively, nonspecific staining was substantially mitigated as we observed correct staining pattern of Synaptophysin labeled with MARS‐dye conjugated secondary antibody in mouse brain (Figure 5c; Figure S11c, Supporting Information).

We next demonstrated the ability of MAGNIFIERS in visualizing nano‐architecture of specific proteins with MARS dyes. By performing immuno‐eprSRS of actinin 4 (ACTN4) in human kidney FFPE tissue with MARS dye conjugated secondary antibody, we observed ultrafine structures of the podocyte foot process by targeting the nitrile mode of MARS2228 (Figure 5d; Figure S12a, Supporting Information), consistent with standard immunofluorescence result.^[^ ^21^ ^]^ The periodic pattern of the foot processes, which have a width ≈200 nm, was clearly visualized in the line plot (Figure 5e; Figure S12a, Supporting Information). Going beyond immunolabeling, other labeling approaches such as glycoprotein labeling with lectins (Figure 5f) and pan‐proteome staining^[^ ^32^ ^]^ with NHS ester or maleimide functionalized MARS probes (Figure 5g; Figure S12b–d, Supporting Information) are generally viable to MAGNIFIERS in a 3D fashion (Videos S3 and S4, Supporting Information ). Of note, MARS dye staining and imaging does not compromise label‐free imaging of endogenous biomolecules, as shown in Figure 5f. Collectively, these results demonstrated the applicability of Raman dye imaging with high molecular specificity in MAGNIFIERS.

### 2.6 One‐Shot Highly Multiplexed Nanoscale Imaging

We next set out to showcase the one‐shot highly‐multiplexed nanoscale imaging capability of MAGNIFIERS in the mouse brain tissue section. To pursue better image contrast, we employ a simple but effective signal amplification strategy by using recurrent staining of MARS‐conjugated secondary antibodies. In detail, as illustrated in Figure S13a (Supporting Information), two different secondary antibodies, each of which binds to the other, will be applied alternatively after regular immunostaining process. Since post‐expansion staining was utilized, the apparent size of the antibody was much smaller compared to the targeted structure in expanded samples. Therefore, recurrent staining rounds were unlikely to affect the interpretation of the shape and size of targeted proteins, as has been calibrated through standard immunofluorescence.^[^ ^53^ ^]^ We tested this amplification method on mouse brain tissue via targeting synaptophysin, a protein found in synaptic vesicles and commonly used as a marker of pre‐synaptic structures.^[^ ^54^ ^]^ After additional 4‐round recurrent staining of MARS2228‐conjugated secondary antibodies, we observed substantial signal enhancement without noticeable background (Figure S13b,c, Supporting Information). Quantitatively, epr‐SRS signal of the skeletal C═C mode of MARS2228 increased by 1.7‐fold and 2.8‐fold after 2‐round and 4‐round amplification, respectively (Figure S13d, Supporting Information).

**Figure 6:**
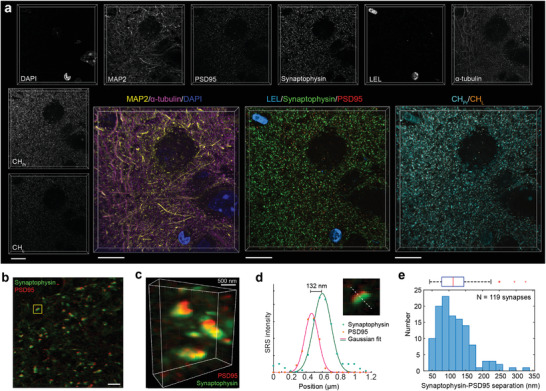
One‐shot highly multiplexed nanoscale imaging. a) One‐shot 8‐plex 3D‐rendered nanoscale imaging of post‐expansion mouse brain slice. Fluorescence: DAPI (total DNA), microtubule associated protein 2 (MAP2, Cy3), *L. Esculentum* lectin (LEL‐DyLight 649, blood vessels). SRS: Synaptophysin (MARS2228, synapse vesicles), *α*‐tubulin (MARS2176), postsynaptic density protein 95 (PSD95, MARS2147, post‐synaptic membrane), CH_Pr_ and CH_L_. Voxel size is 0.318  × 0.318  × 1 µm in post‐expansion distance. b) Single‐plane image of overlayed synaptophysin and PSD95 channels. c) Zoom‐in of 3D‐rendered overlayed synaptophysin and PSD95 image. Yellow box outlined zoom‐in image in (d) for separation distance analysis. d) A representative image of single synapses and corresponding plot of the staining intensity of PSD95 and synaptophysin along the dotted line. Solid lines, Gaussian fits. e) Statistical analysis of Synaptophysin‐PSD95 separations (*n* = 119 synapses). Above is the box plot of the separation distances. Images were acquired with a 25× objective in 1× PBS (4.5‐fold expansion). Scale bars (in biological scales), 10 µm in (a), 2 µm in (b), 500 nm in (c).

With this strategy, we acquired three epr‐SRS channels of synaptophysin, *α*‐tubulin and postsynaptic density‐95 (PSD95) each with additional 4‐round staining in expanded mouse brain tissue. Fluorescence channels are also readily retained for nucleus (DAPI), microtubule associated protein 2 (MAP2), and *Lycopersicon esculentum* (tomato) lectin (LEL). Adding up the chemical imaging of CH_Pr_ and CH_L_ channels, we achieved a total of eight channels in volume in a single round (**Figure** **6**a), with an expansion of ≈4.5‐fold in each dimension (in 1× PBS, effective resolution of ≈73 nm). Such a one‐shot method bypasses the error‐prone registration procedure and is particularly favorable for precise profiling of nano‐architectures in thick volumes.

To showcase the nanoscale spatial precision, we focused on two synaptic marker proteins of synaptophysin (marker of synaptic vesicles) and PSD95. The post‐ExM image explicitly revealed synaptophysin juxtaposed with PSD95 puncta (Figure 6b,c; Video S5, Supporting Information). The separation distance between synaptophysin‐PSD95 pairs was measured after the Gaussian fitting of the signal intensity along the line perpendicular to the synaptic cleft (Figure 6d). The synaptophysin‐PSD95 separation was measured as 118 ± 52 nm (Figure 6e, mean ± s.d., *n* = 119 synapses) in MAGNIFIERS. Our result is close to the prior measurement of separations between postsynaptic densities and synaptic vesicles of ≈150 nm using super‐resolution fluorescence imaging.^[^ ^54^ ^]^ To our best knowledge, vibrational imaging has not been applied to visualize individual synaptic features before. Overall, we expect that MAGNIFIERS will provide a parallel and scalable way to interrogate nano‐architectures in 3D spatial context with molecular specificity.

## 3 Discussion

SRS microscopy and ExM, two emerging techniques, have been synergistically integrated in MAGNIFIERS, providing niches that are complementary to existing SRS or ExM imaging alone. On one side, MAGNIFIERS offers a simple and general way to achieve super‐resolution Raman imaging; on the other side, it complements the prevalent fluorescence‐based nanoscopy. Leveraging the wide‐ranging capabilities of SRS microscopy, we have demonstrated three classes of applications: label‐free chemical imaging, metabolic imaging, and multiplexed imaging with nanoscale precision (Figure 1a). For chemical imaging (Figures 2 and 3), MAGNIFIERS provides label‐free access to visualize proteins, lipids, and DNA biomolecules with effective resolution up to ≈41 nm. We have demonstrated this utility in varied biologically meaningful scenarios, such as visualization of the ultrastructure of basal bodies and investigation of the chemical composition of EVs in human lung organoids. We foresee that MAGNIFIERS will contribute single‐vesicle level understanding of EVs in vivo. For metabolic imaging (Figure 4), we studied protein synthesis and turnover on protein aggregates using deuterated amino acid labeling. In multiplexed imaging with Raman dyes (Figures 5 and 6), MAGNIFIERS offers a one‐shot optical approach to simultaneously map multiple targets in 3D in mouse brain sections. Finally, these applications are additive. For instance, chemical imaging is compatible with imaging of metabolic activities or post‐expansion Raman dye imaging, providing a comprehensive view of the chemical content of the sample.

An interesting technique called VISTA combines label‐free SRS imaging of protein with a special ExM protocol named magnified analysis of the proteome (MAP).^[^ ^29^, ^55^ ^]^ Compared to VISTA, MAGNIFIERS demonstrates expansion SRS microscopy on a much broader range of biological specimens.^[^ ^30^ ^]^ Foremost, unlike VISTA that requires the acrylamide co‐fixation,^[^ ^26^, ^29^ ^]^ MAGNIFIERS is not limited to soft tissue and features general sample compatibility across a broad range of tissue types and fixation methods. We have shown that mechanically tough tissues such as FFPE human kidney can be robustly expanded and imaged by MAGNIFIERS, thus making it readily applicable to the vast archive of human specimens. Moreover, by virtue of more effective preservation of biomolecules (near 100% for protein) and tunable expansion level, our approach offers better sensitivity. This not only enables nanoscale label‐free imaging of protein, nucleic acid, and lipids, but also allows us to open up nanoscale metabolic imaging and highly multiplexed Raman dye imaging, which generally have weaker signals than label‐free applications. Last, MAGNIFIERS provides finer spatial resolution through leveraging excellent reversible linear expansion of ≈7.2‐fold.

Future improvements and opportunities reside in several fronts. First, for label‐free imaging, replacing the gel with deuterated monomers and expanding them in heavy water will be favorable to visualize small signals such as lipids and DNA. Second, the full expansion potential of the MAGNIFY gel has not been thoroughly utilized. To pursue higher effective resolution, femtosecond pulse excitation could be a direct way to enhance broadband SRS signals such as C—H and C—D bonds by an order of magnitude.^[^ ^56^ ^]^ In this way, full expansion of ≈10‐fold in water will be viable for label‐free protein imaging. Moreover, for Raman dye imaging, other DNA‐based signal amplification techniques such as immuno‐SABER^[^ ^47^ ^]^ and isHCR^[^ ^57^ ^]^ are expected to boost the signal more efficiently. Particularly, these amplification strategies will further increase the multiplexity with a practical goal of over 20 channels.^[^ ^7^ ^]^ Third, specimens become optically transparent after expansion and thus MAGNIFIERS has promising applications for thick specimens of millimeter scales, considering successful precedents in integrating tissue clearing with label‐free SRS^[^ ^58^ ^]^ or highly‐multiplexed Raman dye imaging.^[^ ^11^ ^]^ This would open exciting applications to probe interactions between ultrastructure with high content over a long distance, such as with neuronal tracing. Finally, while SRS may have low throughput for profiling large‐volume specimens at high precision, the reversible and size‐controllable features of the MAGNIFY gel provide good flexibility to combine large volume profiling and small region‐of‐interest interrogation in the same sample.

## Conflict of Interest

The authors declare the following competing financial interest(s): Y.Z., A.K., and F.F. are inventors on several inventions related to ExM methods.

## Author Contributions

L.S. performed SRS imaging experiments and analyzed related data. A.K., B.R.G., F.F., and Y.Z. performed all expansion experiments and related characterizations. Z.C. contributed to image processing of multiplex SRS images. P.W. and X.R. prepared lung organoid samples. Y.M. synthesized MARS probes. L.S., Y.Z., and W.M. conceived the concept and wrote the manuscript with input from all authors. Y.Z. and W.M. supervised the project.

## Supporting information

Supporting Information

Supplemental Video 1

Supplemental Video 2

Supplemental Video 3

Supplemental Video 4

Supplemental Video 5

## Data Availability

The data that support the findings of this study are available in the supplementary material of this article.

## References

[1] C. W. Freudiger , W. Min , B. G. Saar , S. Lu , G. R. Holtom , C. He , J. C. Tsai , J. X. Kang , X. S. Xie , Science 2008, 322, 1857. 19095943 10.1126/science.1165758 PMC3576036

[2] F. Hu , L. Shi , W. Min , Nat. Methods 2019, 16, 830. 31471618 10.1038/s41592-019-0538-0

[3] J.‐X. Cheng , X. S. Xie , Science 2015, 350, aaa8870. 26612955 10.1126/science.aaa8870

[4] L. Wei , F. Hu , Z. Chen , Y. Shen , L. Zhang , W. Min , Acc. Chem. Res. 2016, 49, 1494. 27486796 10.1021/acs.accounts.6b00210 PMC5704954

[5] L. Wei , Y. Shen , F. Xu , F. Hu , J. K. Harrington , K. L. Targoff , W. Min , ACS Chem. Biol. 2015, 10, 901. 25560305 10.1021/cb500787b PMC4610303

[6] L. Wei , Z. Chen , L. Shi , R. Long , A. V. Anzalone , L. Zhang , F. Hu , R. Yuste , V. W. Cornish , W. Min , Nature 2017, 544, 465. 28424513 10.1038/nature22051 PMC5939925

[7] F. Hu , C. Zeng , R. Long , Y. Miao , L. Wei , Q. Xu , W. Min , Nat. Methods 2018, 15, 194. 29334378 10.1038/nmeth.4578 PMC5831481

[8] Y. Miao , L. Shi , F. Hu , W. Min , Phys. Biol. 2019, 16, 041003. 30870829 10.1088/1478-3975/ab0fcd

[9] L. Wei , W. Min , J. Phys. Chem. Lett. 2018, 9, 4294. 30001137 10.1021/acs.jpclett.8b00204 PMC6077771

[10] Y. Miao , N. Qian , L. Shi , F. Hu , W. Min , Nat. Commun. 2021, 12, 4518. 34312393 10.1038/s41467-021-24855-6 PMC8313527

[11] L. Shi , M. Wei , Y. Miao , N. Qian , L. Shi , R. A. Singer , R. K. P. Benninger , W. Min , Nat. Biotechnol. 2022, 40, 364. 34608326 10.1038/s41587-021-01041-z PMC8930416

[12] F.‐K. Lu , S. Basu , V. Igras , M. P. Hoang , M. Ji , D. Fu , G. R. Holtom , V. A. Neel , C. W. Freudiger , D. E. Fisher , X. S. Xie , Proc. Natl. Acad. Sci. USA 2015, 112, 11624. 26324899 10.1073/pnas.1515121112 PMC4577158

[13] A. Gasecka , A. Daradich , H. Dehez , M. Piché , D. Côté , Opt. Lett. 2013, 38, 4510. 24177132 10.1364/ol.38.004510

[14] W. R. Silva , C. T. Graefe , R. R. Frontiera , ACS Photonics 2016, 3, 79.

[15] L. Gong , W. Zheng , Y. Ma , Z. Huang , Nat. Photonics 2020, 14, 115.

[16] L. Gong , W. Zheng , Y. Ma , Z. Huang , Phys. Rev. Appl. 2019, 11, 034041.

[17] K. Watanabe , A. F. Palonpon , N. I. Smith , L.‐D. Chiu , A. Kasai , H. Hashimoto , S. Kawata , K. Fujita , Nat. Commun. 2015, 6, 10095. 26626144 10.1038/ncomms10095 PMC4686755

[18] A. P. Olson , C. T. Ertsgaard , S. N. Elliott , N. C. Lindquist , ACS Photonics 2016, 3, 329.

[19] A. T. Wassie , Y. Zhao , E. S. Boyden , Nat. Methods 2019, 16, 33. 30573813 10.1038/s41592-018-0219-4 PMC6373868

[20] F. Chen , P. W. Tillberg , E. S. Boyden , Science 2015, 347, 543. 25592419 10.1126/science.1260088 PMC4312537

[21] Y. Zhao , O. Bucur , H. Irshad , F. Chen , A. Weins , A. L. Stancu , E.‐Y. Oh , M. Distasio , V. Torous , B. Glass , I. E. Stillman , S. J. Schnitt , A. H. Beck , E. S. Boyden , Nat. Biotechnol. 2017, 35, 757. 28714966 10.1038/nbt.3892 PMC5548617

[22] P. W. Tillberg , F. Chen , K. D. Piatkevich , Y. Zhao , C.‐C. Yu , B. P. English , L. Gao , A. Martorell , H.‐J. Suk , F. Yoshida , E. M. Degennaro , D. H. Roossien , G. Gong , U. Seneviratne , S. R. Tannenbaum , R. Desimone , D. Cai , E. S. Boyden , Nat. Biotechnol. 2016, 34, 987. 27376584 10.1038/nbt.3625 PMC5068827

[23] T. J. Chozinski , A. R. Halpern , H. Okawa , H.‐J. Kim , G. J. Tremel , R. O. L. Wong , J. C. Vaughan , Nat. Methods 2016, 13, 485. 27064647 10.1038/nmeth.3833 PMC4929147

[24] F. Chen , A. T. Wassie , A. J. Cote , A. Sinha , S. Alon , S. Asano , E. R. Daugharthy , J.‐B. Chang , A. Marblestone , G. M. Church , A. Raj , E. S. Boyden , Nat. Methods 2016, 13, 679. 27376770 10.1038/nmeth.3899 PMC4965288

[25] S. Truckenbrodt , M. Maidorn , D. Crzan , H. Wildhagen , S. Kabatas , S. O. Rizzoli , EMBO Rep. 2018, 19, e45836. 29987134 10.15252/embr.201845836 PMC6123658

[26] T. Ku , J. Swaney , J.‐Y. Park , A. Albanese , E. Murray , J. H. Cho , Y.‐G. Park , V. Mangena , J. Chen , K. Chung , Nat. Biotechnol. 2016, 34, 973. 27454740 10.1038/nbt.3641 PMC5070610

[27] J. Park , S. Khan , D. H. Yun , T. Ku , K. L. Villa , J. E. Lee , Q. Zhang , J. Park , G. Feng , E. Nedivi , K. Chung , Sci. Adv. 2021, 7, eabf6589. 34767453 10.1126/sciadv.abf6589 PMC8589305

[28] H.‐E. Park , D. Choi , J. S. Park , C. Sim , S. Park , S. Kang , H. Yim , M. Lee , J. Kim , J. Pac , K. Rhee , J. Lee , Y. Lee , Y. Lee , S.‐Y. Kim , Adv. Sci. 2019, 6, 1901673. 10.1002/advs.201901673 PMC6864509 31763149

[29] C. Qian , K. Miao , L.‐E. Lin , X. Chen , J. Du , L. Wei , Nat. Commun. 2021, 12, 3648. 34131146 10.1038/s41467-021-23951-x PMC8206358

[30] A. Klimas , B. Gallagher , P. Wijesekara , S. Fekir , D. Stolz , F. Cambi , S. Watkins , A. Barth , C. Moore , X. Ren , Y. Zhao , Nat. Portfolio 2021, 10.21203/rs.3.rs-858006/v1.

[31] C. Mao , M. Y. Lee , J.‐R. Jhan , A. R. Halpern , M. A. Woodworth , A. K. Glaser , T. J. Chozinski , L. Shin , J. W. Pippin , S. J. Shankland , J. T. C. Liu , J. C. Vaughan , Sci. Adv. 2020, 6, eaba4542. 32518827 10.1126/sciadv.aba4542 PMC7253160

[32] O. M'saad , J. Bewersdorf , Nat. Commun. 2020, 11, 3850. 32737322 10.1038/s41467-020-17523-8 PMC7395138

[33] M. Waldman , R. J. Crew , A. Valeri , J. Busch , B. Stokes , G. Markowitz , V. D'agati , G. Appel , Clin. J. Am. Soc. Nephrol. 2007, 2, 445. 17699450 10.2215/CJN.03531006

[34] M. C. Burke , F.‐Q. Li , B. Cyge , T. Arashiro , H. M. Brechbuhl , X. Chen , S. S. Siller , M. A. Weiss , C. B. O'connell , D. Love , C. J. Westlake , S. D. Reynolds , R. Kuriyama , K.‐I. Takemaru , J. Cell Biol. 2014, 207, 123. 25313408 10.1083/jcb.201406140 PMC4195830

[35] G. Van Niel , G. D'angelo , G. Raposo , Nat. Rev. Mol. Cell Biol. 2018, 19, 213. 29339798 10.1038/nrm.2017.125

[36] T. Skotland , K. Sandvig , A. Llorente , Prog. Lipid Res. 2017, 66, 30. 28342835 10.1016/j.plipres.2017.03.001

[37] M. Pathan , P. Fonseka , S. V. Chitti , T. Kang , R. Sanwlani , J. Van Deun , A. Hendrix , S. Mathivanan , Nucleic Acids Res. 2019, 47, D516. 30395310 10.1093/nar/gky1029 PMC6323905

[38] R. Kalluri , V. S. Lebleu , Science 2020, 367, eaau6977. 32029601 10.1126/science.aau6977 PMC7717626

[39] F. J. Verweij , L. Balaj , C. M. Boulanger , D. R. F. Carter , E. B. Compeer , G. D'angelo , S. El Andaloussi , J. G. Goetz , J. C. Gross , V. Hyenne , E.‐M. Krämer‐Albers , C. P. Lai , X. Loyer , A. Marki , S. Momma , E. N. M. Nolte‐‘T Hoen , D. M. Pegtel , H. Peinado , G. Raposo , K. Rilla , H. Tahara , C. Théry , M. E. Van Royen , R. E. Vandenbroucke , A. M. Wehman , K. Witwer , Z. Wu , R. Wubbolts , G. Van Niel , Nat. Methods 2021, 18, 1013. 34446922 10.1038/s41592-021-01206-3 PMC8796660

[40] H. Yamakoshi , K. Dodo , A. Palonpon , J. Ando , K. Fujita , S. Kawata , M. Sodeoka , J. Am. Chem. Soc. 2012, 134, 20681. 23198907 10.1021/ja308529n

[41] L. Wei , Y. Yu , Y. Shen , M. C. Wang , W. Min , Proc. Natl. Acad. Sci. USA 2013, 110, 11226. 23798434 10.1073/pnas.1303768110 PMC3710790

[42] L. Shi , X. Liu , L. Shi , H. T Stinson , J. Rowlette , L. J. Kahl , C. R. Evans , C. Zheng , L. E. P. Dietrich , W. Min , Nat. Methods 2020, 17, 844. 32601425 10.1038/s41592-020-0883-z PMC7396315

[43] C. Huan , C. Wei‐na , J. Sunggoan , R. Sarah , M. Stuart , M. Bronwen , Curr. Alzheimer Res. 2012, 9, 5.22329649

[44] M. Lu , L. Banetta , L. J. Young , E. J. Smith , G. P. Bates , A. Zaccone , G. S. Kaminski Schierle , A. Tunnacliffe , C. F. Kaminski , J. Biol. Chem. 2019, 294, 257. 30401748 10.1074/jbc.RA118.003500 PMC6322900

[45] S. J. Sahl , L. E. Weiss , W. C. Duim , J. Frydman , W. E. Moerner , Sci. Rep. 2012, 2, 895. 23193437 10.1038/srep00895 PMC3508451

[46] M. Klevanski , F. Herrmannsdoerfer , S. Sass , V. Venkataramani , M. Heilemann , T. Kuner , Nat. Commun. 2020, 11, 1552. 32214101 10.1038/s41467-020-15362-1 PMC7096454

[47] S. K. Saka , Y. Wang , J. Y. Kishi , A. Zhu , Y. Zeng , W. Xie , K. Kirli , C. Yapp , M. Cicconet , B. J. Beliveau , S. W. Lapan , S. Yin , M. Lin , E. S. Boyden , P. S. Kaeser , G. Pihan , G. M. Church , P. Yin , Nat. Biotechnol. 2019, 37, 1080. 31427819 10.1038/s41587-019-0207-y PMC6728175

[48] S.‐M. Guo , R. Veneziano , S. Gordonov , L. Li , E. Danielson , K. Perez De Arce , D. Park , A. B. Kulesa , E.‐C. Wamhoff , P. C. Blainey , E. S. Boyden , J. R. Cottrell , M. Bathe , Nat. Commun. 2019, 10, 4377. 31558769 10.1038/s41467-019-12372-6 PMC6763432

[49] S. S. Agasti , Y. Wang , F. Schueder , A. Sukumar , R. Jungmann , P. Yin , Chem. Sci. 2017, 8, 3080. 28451377 10.1039/c6sc05420j PMC5380918

[50] H. Fujioka , J. Shou , R. Kojima , Y. Urano , Y. Ozeki , M. Kamiya , J. Am. Chem. Soc. 2020, 142, 20701. 33225696 10.1021/jacs.0c09200

[51] D. Sarkar , J. Kang , A. T. Wassie , M. E. Schroeder , Z. Peng , T. B. Tarr , A. Tang , E. Niederst , J. Z. Young , L. Tsai , T. A. Blanpied , E. S. Boyden , bioRxiv, 2020, 10.1101/2020.08.29.273540, 2020.2008.2029.273540.

[52] E A. Susaki , C. Shimizu , A. Kuno , K. Tainaka , X. Li , K. Nishi , K. Morishima , H. Ono , K. L. Ode , Y. Saeki , K. Miyamichi , K. Isa , C. Yokoyama , H. Kitaura , M. Ikemura , T. Ushiku , Y. Shimizu , T. Saito , T. C. Saido , M. Fukayama , H. Onoe , K. Touhara , T. Isa , A. Kakita , M. Shibayama , H. R. Ueda , Nat. Commun. 2020, 11, 1982. 32341345 10.1038/s41467-020-15906-5 PMC7184626

[53] Y. Cho , J. Seo , Y. Sim , J. Chung , C. E. Park , C. G. Park , D. Kim , J.‐B. Chang , Nanoscale 2020, 12, 23506. 33215627 10.1039/d0nr05800a

[54] A. D. Sauerbeck , M. Gangolli , S. J. Reitz , M. H. Salyards , S. H. Kim , C. Hemingway , M. Gratuze , T. Makkapati , M. Kerschensteiner , D. M. Holtzman , D. L. Brody , T T. Kummer , Neuron 2020, 107, 257. 32392471 10.1016/j.neuron.2020.04.012 PMC7381374

[55] L.‐E. Lin , K. Miao , C. Qian , L. Wei , Analyst 2021, 146, 4135. 33949430 10.1039/d1an00060h PMC8238904

[56] D. Zhang , M. N. Slipchenko , J.‐X. Cheng , J. Phys. Chem. Lett. 2011, 2, 1248. 21731798 10.1021/jz200516n PMC3124560

[57] R. Lin , Q. Feng , P. Li , P. Zhou , R. Wang , Z. Liu , Z. Wang , X. Qi , N. Tang , F. Shao , M. Luo , Nat. Methods 2018, 15, 275. 29481551 10.1038/nmeth.4611

[58] M. Wei , L. Shi , Y. Shen , Z. Zhao , A. Guzman , L. J. Kaufman , L. Wei , W. Min , Proc. Natl. Acad. Sci. USA 2019, 116, 6608. 30872474 10.1073/pnas.1813044116 PMC6452712

