# Supplementary material for: Super‐Resolution Vibrational Imaging Using Expansion Stimulated Raman Scattering Microscopy
Source: Adv Sci (Weinh). 2022 May 6;9(20):2200315. doi: 10.1002/advs.202200315 (PMC9284179; doi:10.1002/advs.202200315)
Supplement: Supplementary file 1 — Supporting Information [file ADVS-9-2200315-s005.pdf]

ADVANCED SCIENCE
Open Access

Supporting Information

for *Adv. Sci.*, DOI 10.1002/advs.202200315

Super-Resolution Vibrational Imaging Using Expansion Stimulated Raman Scattering Microscopy

Lixue Shi, Aleksandra Klimas, Brendan Gallagher, Zhangyu Cheng, Feifei Fu, Piyumi Wijesekara, Yupeng Miao, Xi Ren, Yongxin Zhao and Wei Min**

Supplementary Information for

Super-resolution vibrational imaging using expansion stimulated Raman scattering microscopy

Lixue Shi[1], Aleksandra Klimas[2], Brendan Gallagher[2], Zhangyu Cheng[2], Feifei Fu[2], Piyumi Wijesekara[3], Yupeng Miao[1], Xi Ren[3], Yongxin Zhao[2*], Wei Min[1,4*]

[1]Department of Chemistry, Columbia University, New York, NY, USA.
[2]Department of Biological Sciences, Carnegie Mellon University, Pittsburgh, PA, USA.
[3]Department of Biomedical Engineering, Carnegie Mellon University, Pittsburgh, PA, USA.
[4]Kavli Institute for Brain Science, Columbia University, New York, NY, USA.
[*]Corresponding author: wm2256@columbia.edu, yongxinz@andrew.cmu.edu

This file includes:

Methods
Supplementary Figures 1-13
Supplementary Tables 1-3
Supplementary Videos 1-5
References

Methods

Materials and human samples. Detailed information regarding all reagents and equipment was described in Supplementary Table 1. MARS dyes and their derivatives (NHS-ester- and maleimide-functionalized) were synthesized as previously reported[1]. The human pathology specimens were purchased from US Biomax catalog numbers HuFPT072 (normal human kidney cortex), HuFPT015 (normal human hippocampus), HuFPT082 (normal human spleen).

Mouse sample preparation. Animal experimental protocol (AC-AABD1552) was approved by the Institutional Animal Care and Use Committee (IACUC) at Columbia University. All experiments using mice were conducted in strict adherence to the ethical regulations of Columbia University IACUC. Wild type male and female mice (C57BL/6, ~5 weeks old, Jackson Lab) were fully anesthetized using isoflurane, then sacrificed with cervical displacement and immediately perfused with 4% paraformaldehyde (PFA) in PBS transcranially. The brain, kidney, liver and pancreas were extracted and fixed in 4% PFA in PBS at 4 °C for 24 h. After that, the collected organs were immersed in PBS at 4 °C for 24 h to remove PFA. The organ was embedded in 7% agarose gel and sectioned into 40 µm thick coronal slices using a vibratome (VT1000S, Leica). Agarose was removed by a tweezer after sectioning.

Culture of airway basal cells. The normal human bronchial epithelial (NHBE) cultured in 804G-conditioned medium coated culture vessels in bronchial epithelial cell growth medium (BEGM) supplemented with 1uM A8301, 5uM Y27632, 0.2uM of DMH-1, and 0.5uM of CHIR99021[2] at 37°C with 5% CO_2.

Differentiation of airway basal cells into airway organoids. A 96-well tissue culture plate was coated with 40% (vol/vol) growth factor reduced (GFR) Matrigel in PneumaCult™-ALI Maintenance Medium. The NHBEs were resuspended in 40% (vol/vol) GFR Matrigel in PneumaCult™-ALI Maintenance Medium and added to the coated wells. 100µL PneumaCult™-ALI Maintenance Medium was placed in the wells and changed every other day. The cultures were maintained at 37°C with 5% CO_2 for 21 days.

Deuterated amino acids labeling to study huntingtin protein aggregation. HeLa cells (ATCC CCL-2) were cultured in DMEM (11965, ThermoFisher) supplemented with 10% FBS (10082, ThermoFisher) and 1% penicillin/streptomycin (1514, ThermoFisher). Cells were first seeded onto a clean coverslip and cultured with their usual culture media for 24 h, followed by transfection with 200 ng Htt-Q74 (Addgene, #40262, tagged with EGFP) plasmid using Lipofectamine™ 3000 transfection reagent in regular DMEM or CD-DMEM (see previous report[3] for recipe). After 48 hr incubation, cells were washed with DPBS and fixed with 4% PFA for 15 min. Cells were then expanded following the protocol described above.

Protein-MARS probe conjugation. NHS-ester-functionalized MARS probes were stored at a concentration of 10 mM in DMSO under -20 ℃, protecting from light and moisture. To perform protein-dye conjugation, dye solutions were first diluted in DMSO to a concentration of 2 mg/mL. Conjugation buffer was prepared as 0.1 M $NaHCO_3$ in PBS buffer with pH adjusted to 8.3. Highly cross-adsorbed secondary antibodies were buffer exchanged and concentrated to 2 mg/mL in the conjugation buffer. A 50 µL dye-NHS solution was slowly added to a 0.5 mL secondary antibody solution under stirring. For primary antibody labeling, the protein concentration was adjusted to 1 mg/ml and the molar ratio of dye/protein was usually 10-15. Lectins were first dissolved in the conjugation buffer as 2 mg/ml. For *Lycopersicon Esculentum* Lectin labeling, a 25 µL 2 mg/ml dye-NHS solution was added to a 0.5 mL 2 mg/ml LEL solution. Reactions were all incubated at room temperature for 1 h under constant mild stirring. Labeled proteins were further separated from unreacted dyes by gel

permeation chromatography using Sephadex™ G-25 (G25150 SIGMA) resins with a column of 1-cm diameter and over 12-cm length. Purified protein solution was centrifuged to remove potential precipitates and further concentrated with Amicon® Ultra Centrifugal Filters (UFC501096, EMD, Millipore). A final concentration of ~2 mg/mL protein solution (for secondary antibodies and lectins) were prepared in stocking buffer (30% glycerol and 5 mM sodium azide in PBS) and stored at -20 °C. The degree of labeling (DOL, i.e. dye-to-protein ratio) were measured with UV-Vis spectrum using Tecan Infinite 200 Reader with a NanoQuant Plate. DOL on secondary antibodies is about 3.

Stimulated Raman scattering (SRS) and fluorescence integrated imaging platform. SRS and fluorescence (both confocal and two-photon) imaging were performed on an inverted laser scanning microscope (Olympus FV1200) using a 25× water-immersion objective lens (Olympus XLPlan N, 1.05 NA, MP, WD = 2 mm) or a 60× IR-coated water-immersion objective lens (Olympus UPlanApo/IR, 1.2 NA).

For SRS imaging, two synchronized 6-ps lasers (called pump and Stokes beams) with 80-MHz repetition rate are provided by a picoEmerald system from APE (Applied Physics & Electronic, Inc.). Pump beam is tunable from 720-990 nm through both temperature control of the nonlinear crystal and a Lyot filter. Stokes beam is fixed at 1064.2 nm. The intensity of the Stokes beam was modulated sinusoidally by a built-in electro-optic modulator (EOM) at 8 MHz with a modulation depth of more than 90%. Spatially and temporally-overlapped pump and Stokes beams were coupled into the laser-scanning microscope. After passing through the specimens, forward-going pump and Stokes beams were collected with an IR-coated oil condenser (1.4 NA, Olympus). Stokes beam were completely filtered with two high-optical-density bandpass filter (890/220 CARS, Chroma Technology) and transmitted pump beam was detected by a large-area (10 mm×10 mm) Si photodiode (FDS1010, Thorlabs). The output current of the photodiode was then sent to a fast lock-in amplifier (HF2LI, Zurich Instruments) for signal demodulation. For C-H and C-D bonds, the laser power was set as P_{pump}=67-100 mW, P_{Stokes}=100-150 mW, and the pixel dwell time is 60-80 μs and the corresponding time constant of the lock-in amplifier is 30-40 μs. For eprSRS imaging of MARS dyes, the laser power was set as P_{pump}=17 mW, P_{Stokes}=67 mW; and images were generated through Kalman filtering of 10-30 serial frames with the pixel dwell time of 4 μs, and the time constants of lock-in amplifier were chosen as 2-4 μs. For volumetric imaging, the step size in z was 1-2 μm.

For two-photon fluorescence, DAPI dye was excited by the SRS pump laser at 760 nm. The backward fluorescence was detected after passing through a 690-nm short-pass filter, reflected by a 570-nm long-pass dichroic with a collection band of 410-490 nm. For confocal fluorescence, green channel is excited by argon laser (488 nm) with a collection band of 505-520 nm; red channel is excited by HeNe(G) laser (543 nm) with a collection band of 560-620 nm; far-red channel is excited by LD laser (635 nm) with a collection band of 655-755 nm. Multichannel photomultiplier tube (PMT) was used for fluorescence detection. Pixel dwell time was set as 2-4 μs.

Tissue section recovery and heat treatment. Formalin-fixed paraffin-embedded (FFPE) clinical samples were washed in the following solutions 2 times for 3 minutes each at room temperature (RT): xylene, 100% ethanol, 95% ethanol, 70% ethanol, 50% ethanol, and doubly deionized water. For samples that were stained prior to gelation, tissue slides were placed in 20 mM sodium citrate solution (pH 8) at 100 ºC. The container was transferred to a 60 ºC container for 30 minutes.

Permeabilization of fixed tissues. PFA fixed tissues (mouse brain, liver, kidney; HeLa cells; and lung organoid) were permeabilized for 1 hour with 1% C12E10/1xPBS or 1%PBST at RT prior to gelation with the MAGNIFY protocol.

Protein anchoring with ProExM. A stock solution of Acryloyl-X, s.e.m. (6-((acryloyl)amino)hexanoic acid, succinimidyl ester, AcX) was prepared by dissolving in anhydrous DMSO to a concentration of 10 mg/mL. The solution was then aliquoted and stored in a desiccated environment at −20 °C. Tissue slides were incubated with 0.05 mg/ml AcX diluted in 1x PBS buffer overnight at 4°C.

***In situ* polymer synthesis of samples with ProExM.** A gelling solution based on a modified version of the expansion pathology (ExPath) protocol was used containing 15% (w/v) SA, 5% (w/v) AA, 0.1% (w/v) Bis, 11.7% (w/v) NaCl, and 1× PBS was prepared in water. The chemicals 4HT, TEMED, and APS were added to the gel monomer solution to a final concentration of 0.01% (w/v) 4HT, 0.2% (v/v) TEMED, and 0.2% (w/v) APS. After mixing the solution was vortexed, and tissues were incubated with the gelling solution for 30 minutes at 4 ºC to allow the monomer solution to diffuse into the tissue while preventing premature gelation. A gelling chamber was then constructed around the tissue, consisting of spacers cut from #1.5 cover glass and a glass slide on top. The samples were incubated overnight in a humidified container at 37 ºC to complete gelation.

***In situ* polymer synthesis of samples with MAGNIFY.** A monomer solution made of 4% DMAA (v/v), 34% SA (w/v), 10% AA (w/v), 0.01% Bis (w/v), 1% NaCl (w/v), and 1x PBS or the modified ExPath monomer solution, or a monomer solution made of 8% DMAA (v/v), 30% SA (w/v), 5% AA (w/v), 0.01% Bis (w/v), 1% NaCl (w/v), and 1x PBS was prepared and stored at 4C prior to synthesis. Prior to gelation, the chemicals 4HT, APS, TEMED, and methacrolein were added to a final concentration of 0.2-0.25% (w/v) APS, 0-0.25% (v/v) TEMED, 0.001% 4HT (w/v), and 0.1% (v/v) methacrolein immediately prior to gelation. After mixing the solution was vortexed, and tissues were incubated with the gelling solution for 30 minutes at 4 ºC to allow the monomer solution to diffuse into the tissue while preventing premature gelation. A gelling chamber was then constructed around the tissue, consisting of spacers cut from #1.5 cover glass and a glass slide on top. The samples were incubated overnight in a humidified container at 37 ºC to complete gelation.

Protease digestion of samples. After gelation, blank gel surrounding the tissue was trimmed from the samples. Samples were then incubated in the ExPath homogenization buffer (50 mM Tris (pH = 8), 25 mM EDTA, 0.5% w/v Triton X-100, 0.8M NaCl) with Proteinase K diluted by 1:400 (final concentration 2 units/mL). Mouse brain samples were then homogenized at RT for 1-2h. Homogenized samples were then washed 3 times with 1x PBS at RT.

Sample digestion and expansion with MAGNIFY. After gelation, blank gel surrounding the tissue was trimmed from the samples and tissue was cut into smaller pieces. Samples were then incubated in the homogenization buffer (1-10% w/v SDS, 8M Urea, 25 mM EDTA, 2× PBS, pH 7.5 at RT) for1-48h at 80-95ºC with shaking. Homogenized samples were then washed 3 times with 1x PBS at RT, followed by at least 3 washes in 1% C12E10/1xPBS or 1% PBST at RT or 60 ºC to remove remaining SDS.

Estimation of expansion factor. Expansion factors estimated using average nuclear surface area or by matching features in pre- versus post-expansion images. For average nuclear surface area estimates, Images of DAPI stained samples were obtained using a CFI Plan Apo Lambda 10× (NA 0.45) prior to gelation and after homogenization and expansion. Nuclear surface areas were determined using FIJI/ImageJ. To calculate the linear expansion factor, the square root of the ratio of the average post-expansion nuclear surface area to average pre-expansion surface area was calculated. For specimens with pre-expansion images, immunostained samples were imaged using a CFI Plan Apo Lambda 4× (NA 0.2) and CFI Plan Apo Lambda 10× (NA 0.45) objective. After gelling and homogenization, expanded tissue pieces were imaged using a CFI Plan Apo Lambda 4× (NA 0.2) and a CFI Plan Apo Lambda 10× (NA 0.45) objective. Regions of interest in post-expanded images were matched to pre-

expansion regions of interest and the measurement tool in NIS elements or FIJI/ImageJ was used to measure features sizes in both pre- and post-expansion images.

Measurement of SRS resolution by imaging beads. For the 25× objective (NA=1.05), 120 nm polystyrene Latex beads were first diluted in deionized water by 1:100 with vertexing and sonication. The diluted beads were added to 2.5% agarose at 95 °C on a heat plate. The mixed solution was quickly sealed between a glass slide and a coverslip before gel formation. For the 60× objective (NA=1.2), 100 nm polystyrene Latex beads were resuspended in deionized water by a 1:1000 dilution ratio with vertexing and sonication. Then the beads were sandwiched between a glass slide and a coverslip for one day to settle down. The beads were imaged through probing the 3056 cm^{-1} band under each objective.

CH_L/CH_{Pr}/CH_{DNA} unmixing. A previously reported spectral linear combination algorithm was used to unmix lipids, proteins and DNA signals on the C-H region. Briefly, SRS signature peaks at 2848 cm^{-1}, 2941 cm^{-1} and 2967 cm^{-1} were acquired, which bear the Raman features of C–H bonds in lipids, proteins and DNA, respectively. Following equations used to calculate unmixed CH_L, CH_{Pr} and CH_{DNA} signals. I_{2848}, I_{2941} and I_{2967} are SRS microscopic signal intensities at 2848, 2941 and 2967 cm^{-1}, respectively.

$$\begin{pmatrix} CH_{DNA} \\ CH_{Pr} \\ CH_L \end{pmatrix} = \begin{pmatrix} 1.29 & -0.58 & -0.007 \\ -0.66 & 1.38 & -0.105 \\ 0.14 & -0.93 & 1.09 \end{pmatrix} \begin{pmatrix} I_{2967} \\ I_{2941} \\ I_{2848} \end{pmatrix}$$

Post-expansion immunostaining of FFPE samples and expansion. After homogenization and washing, samples were stained with respective primary antibodies diluted to approximately 1 µg/mL in the staining buffer (9× PBS/10% Triton X-100/10mg/L heparin) overnight at room temperature. Samples were then washed 3 times with the washing buffer (1× PBS/0.1% TritonX-100) at room temperature for at least 10 minutes. Samples were then incubated in the staining buffer with the relevant secondary antibodies diluted to 1-10 µg/mL overnight at RT. Samples were then washed at least 3 times with the staining buffer for at least 30 minutes. After staining, samples were washed in 1× PBS for at least 30 minutes. This was repeated until the sample was fully expanded, at least three exchanges of final imaging solution.
DiD staining. Tissue samples were stained with DiD solution diluted as 1:200 to 1× PBS/0.1% Tween-20 overnight at room temperature. After staining, samples were washed in 1× PBS three times, each for 10 minutes.
LEL staining. Tissue samples were stained with Dylight649-conjugated *Lycopersicon Esculentum* lectin solution diluted to 5-10 µg/mL in 1× PBS overnight at room temperature. After staining, samples were washed in 1× PBS three times, each for 10 minutes.

Brain tissue multicolor imaging.
Staining and imaging. Post-expanded brain samples were stained in 2 ml Eppendorf tubes all at room temperature. Samples were incubated with primary antibodies (rabbit anti-synaptophysin, goat PSD95, mouse a-tubulin, chicken anti-TH, guinea pig anti-MAP2) in 9x PBS/10% TritonX/10mg/L heparin overnight at room temperature, followed by washing at room temperature for 10 min in excess volumes of 1xPBS/0.1% TritonX-100 three times. Samples were then stained with MARS conjugated secondary antibodies for four rounds at a concentration of approximately 10 µg/mL each in the staining buffer (5x PBS/10% TritonX) for 18-24 hrs. In the odd rounds, MARS2228-conjugated donkey anti-rabbit IgG antibody, MARS2176-conjugated rat anti-mouse IgG antibody, MARS2145-conjugated bovine anti-goat IgG were applied. In the even rounds, MARS2228-conjugated rabbit anti-donkey IgG, MARS2176-conjugated mouse anti-rat IgG, MARS2145-conjugated goat anti-bovine IgG were applied. Between

rounds, samples were washed for 1 hr in excess volumes of washing buffer (5x PBS/10% TritonX) three times. After that, samples were stained with Cy3-conjugated donkey anti-chicken IgY, Alexa Fluro488 donkey anti-guinea pig IgG, Dylight649-conjugated *Lycopersicon Esculentum* lectin in 5x PBS. Gels were then washed and expanded in excess volumes of 1x PBS for 1 hr. This step was repeated 3-5 times until complete expansion. Lastly, gels were stained with DAPI in 1x PBS for 1 hr. For SRS imaging, gels were sandwiched in imaging gel (1xPBS/1.5% agarose (w/v)/ 1% propyl gallate (w/v)) between the cover slip and the glass slide.

Data processing. A linear-combination algorithm was applied on the raw volumetric epr-SRS data set to remove potential cross-talks between different channels[4]. Measured signals ($\boldsymbol{S}$) can be expressed as $\boldsymbol{S}=M\boldsymbol{C}$, where $\boldsymbol{C}$ is the MARS probe concentrations and M is a 3×3 matrix determined by Raman cross-sections of MARS probes. MARS probe concentrations were therefore determined using $\boldsymbol{C} = M^{-1}\boldsymbol{S}$. All the images were volume-rendered in the mode of maximal intensity projection using Imaris Viewer (Bitplane).

Synapse quantification. Data of Fig. 6a was used for analysis. Synapses were identified by visual inspection. Candidate instances of closely apposed synaptophysin-stained and PSD-95 stained spots/ribbon were selected with following criteria: synapses were oriented perpendicular to the y-axis; complex assemblies of synapses (e.g., with multiple pre- or post-synaptic terminals) were rejected. For each synapse selected for inclusion in the analysis, an averaged (over a width of up to 8 pixels (~500 nm)) line profile of SRS signal was generated along the y-axis. The resulting intensity distributions were fitted to Gaussian distributions with Matlab 'fit' function. Any synapses with a resulting goodness of fit (i.e. rmsd), for either synaptophysin or PSD95, of less than 0.9 were rejected. The separation was calculated as the distance between the fitted centers of the Gaussian distributions for each synapse.

Statistical analysis.

Statistical analysis was carried out using GraphPad Prism 7. Data are presented as mean±s.d. with statistical significance if required (not significant $P \geq 0.05$, *$P<0.05$, **$P<0.01$, ***$P<0.001$, ****$P<0.0001$). All values of *n* are provided in the figure legends. In the box plot (Fig. 1b, 6e, Supplementary Fig. 9c-d, 13d), the center indicates the median; the bottom and top edges of the box indicate the 25th and 75th percentiles, respectively; the whiskers extend to the minimum and maximum data points; the outliers are plotted individually using the '+' marker symbol.

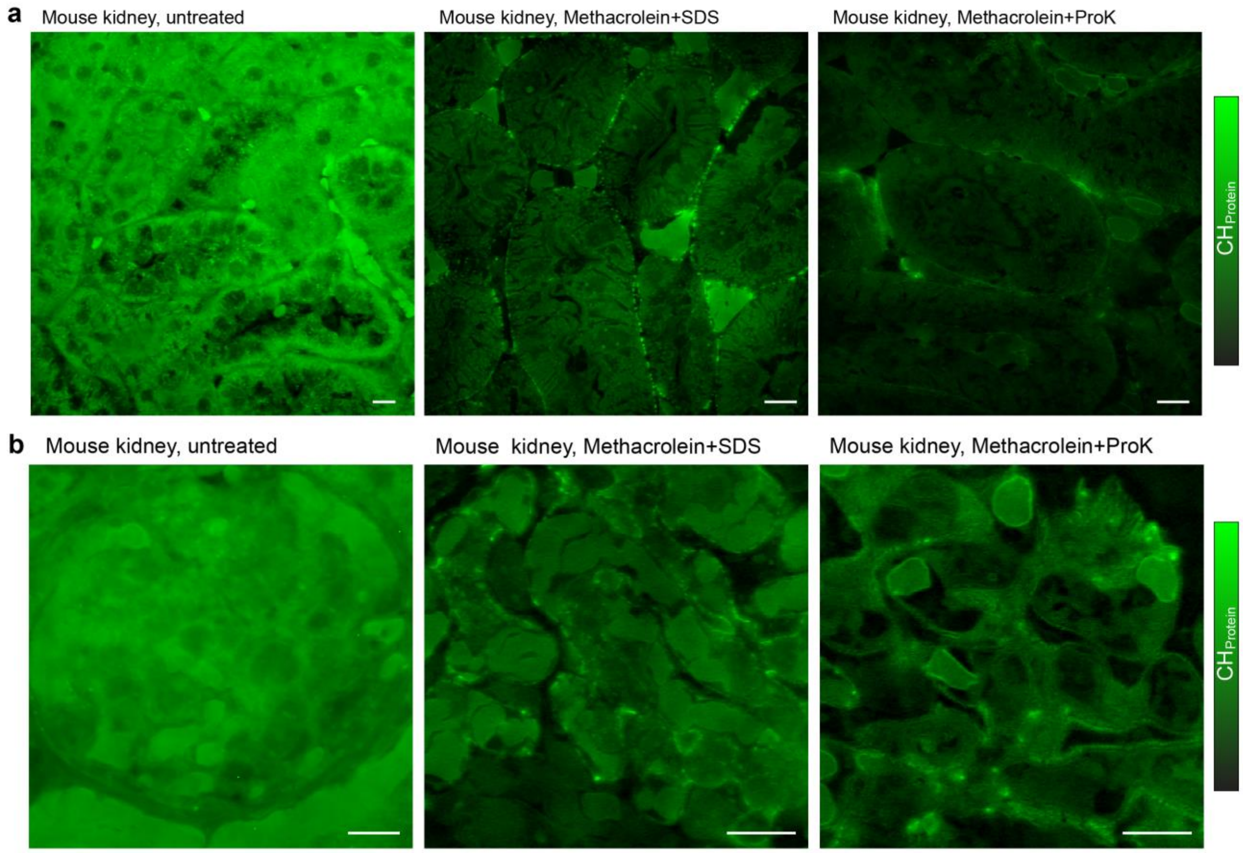

Supplementary Figure 1. Comparison of protein retention on proExM protocol with our protocol. **a**, SRS images of CH_{Pr} of the tubules in the mouse kidney tissues. (left) untreated, (middle) expanded with methacrolein-linking and SDS/urea homogenization, (right) expanded with methacrolein-linking and Proteinase K digestion. **b**, SRS images of unmixed $CH_{protein}$ signal of the glomerulus in the mouse kidney tissues. (left) untreated, (middle) expanded with methacrolein-linking and SDS/urea homogenization, (right) expanded with methacrolein-linking and Proteinase K digestion. Scale bars (in biological scales), 10 μm.

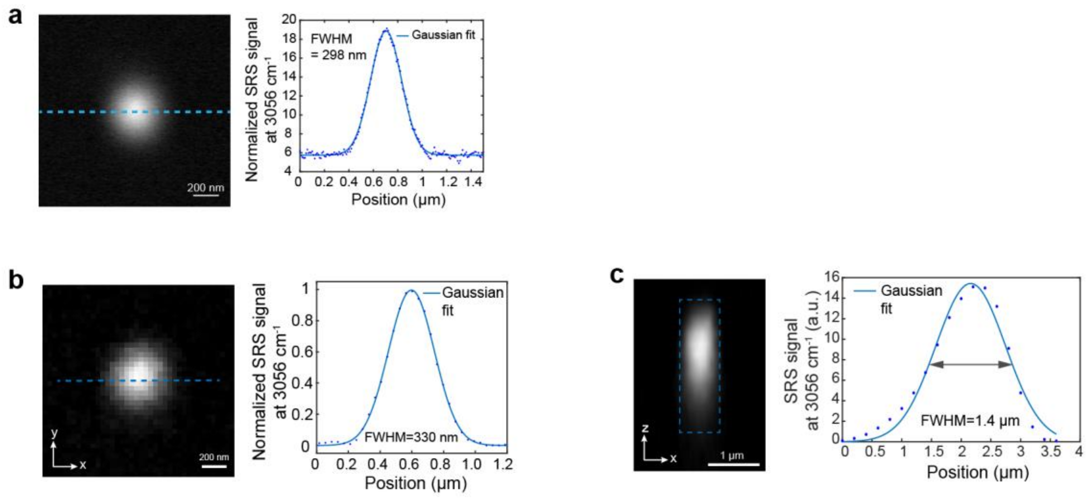

Supplementary Figure 2. Bead quantification of the spatial resolution of SRS microscopy. a, Calibration of lateral resolution of SRS under a 1.2 NA objective with a 100-nm polystyrene bead by measuring the C-H peak at 3056 cm^{-1}. Quantification of (**b**) lateral (after deconvolution) and (**c**) axial resolution of SRS under a 1.05 NA objective with a 120-nm polystyrene bead by measuring the C-H peak at 3056 cm^{-1}. Right graphs are cross-section profiles fitted by the Gaussian function. Scale bars (in biological scales), 200 nm in (**a**, **b**); 1 μm in (**c**);

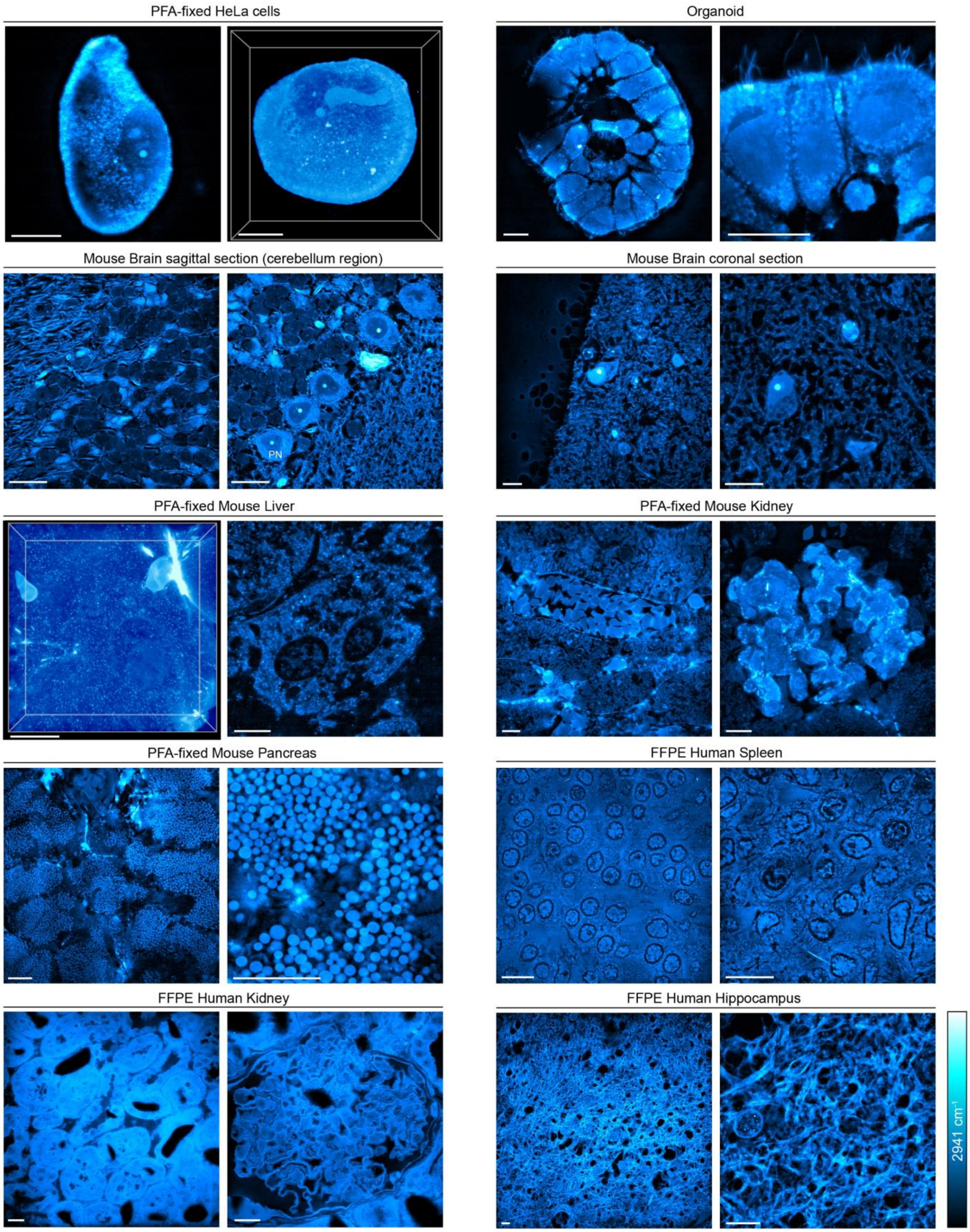

Supplementary Figure 3. MAGNIFlERS has good sample generality. SRS images of CH_3 peak at 2941 cm^{-1} of PFA-fixed HeLa cells, organoids, mouse brain, mouse liver, mouse kidney, mouse pancreas, FFPE human kidney, FFPE human spleen and FFPE human hippocampus tissues. Expansion

factors are 4.5 for HeLa, organoid and mouse brain, 4.3 for mouse kidney, liver and pancreas, 3.8 for FFPE human kidney and FFPE human spleen, 2.0 for FFPE human hippocampus in 1× PBS. Scale bars (in biological scales), 10 μm.

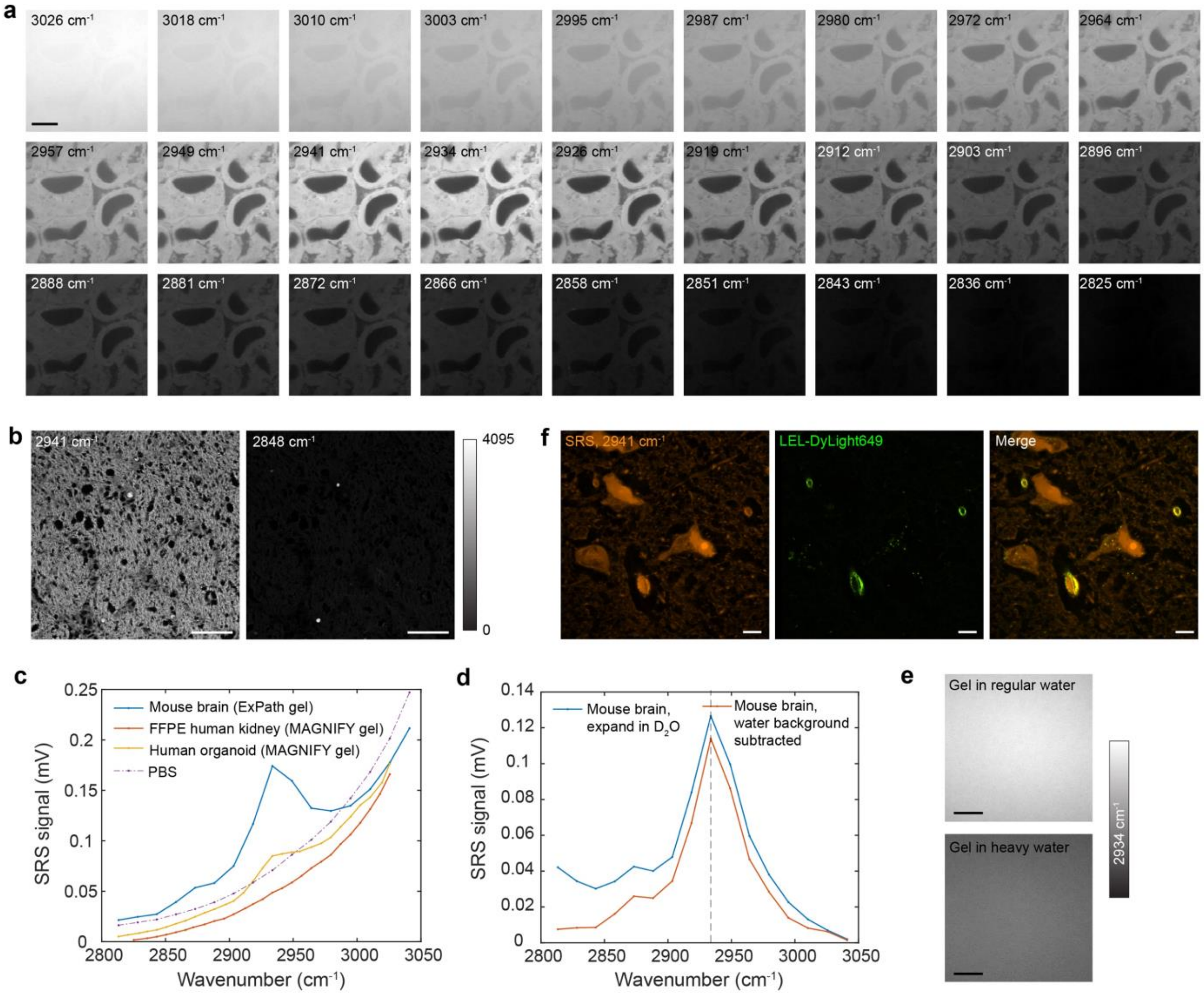

Supplementary Figure 4. Studies on label-free protein imaging in MAGNIFIERS. a, Images of hyperspectral SRS acquisition on the human kidney sample in 1× PBS (2.7-fold expansion). The wavenumbers were labeled at the up-left corner of each image. **b**, SRS images of 2941 cm^{-1} (left) and 2848 cm^{-1} (right) on deparaffinized FFPE human hippocampus tissue. **c**, SRS spectra measured in the non-tissue areas of expanded mouse brain (ExPath gel), FFPE human kidney (MAGNIFY gel), human lung organoid (MAGNIFY gel), and PBS buffer. **d**, Spectral shape of the gel C-H background. **e**, Gel backgrounds are spatially uniform. Up, gel expand in regular buffer; Down, gel background in heavy water replaced buffer. **f**, Expanded mouse brain tissue labeled with *Lycopersicon Esculentum* lectin (LEL)-DyLight 649. Left, SRS image of 2941 cm^{-1}; Right, fluorescence image of blood vessels. In (**c**-**d**), $P_{pump} = 120$ mW, $P_{Stokes} =$ 180 mW. Expanded mouse brain in (**f**) were imaged with a 25× objective in 1× PBS with 4.5-fold expansion. Scale bars (in biological scales), 20 μm in (**a**); 50 μm in (**b, e**); 5 μm in (**f**).

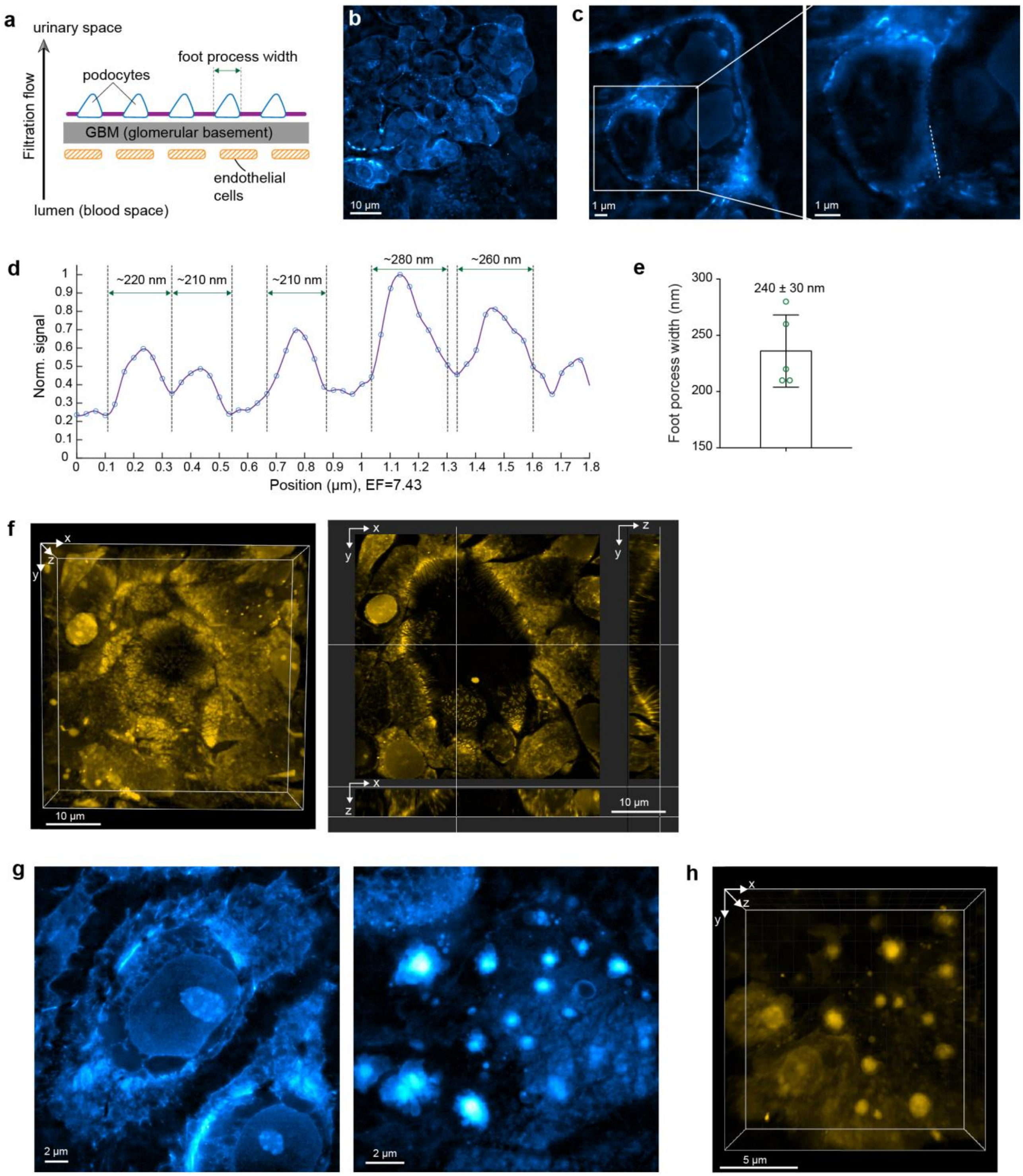

Supplementary Figure 5. CH_3 image of protein reveals ultrafine structures in mouse kidney and human lung organoids. a, Schematics of the structure of three-layered glomerular filtration barrier (GFB). GFB is an intricate structure in kidney glomeruli that builds the selective barrier between bloodstream and the urine. GFB is composed of unique epithelial cells named podocytes which extend many interdigitated foot processes in the glomeruli. **b**-**c**. CH_3 image at 2941 cm^{-1} visualized ultrafine structures of tertiary podocyte foot processes in kidney glomerulus in 1/25× PBS (expansion factor, 7.43). **c**, Right, magnified image of the region outlined by the white box. **d**, Line profile of the dotted line within the right image in (**c**). **e**, Bar plot (mean±s.d.) on foot process width as marked in graph (**d**).

f-**h**, SRS images at 2941 cm^{-1} of expanded human bronchial epithelial cells-derived lung organoids in 1×PBS (expansion factor, 4.5). All results were acquired with a 1.05 NA objective. Scale bars (in biological scales), 10 μm in (**b**, **f**); 1 μm in (**c**); 2 μm in (**g**); 5 μm in (**h**).

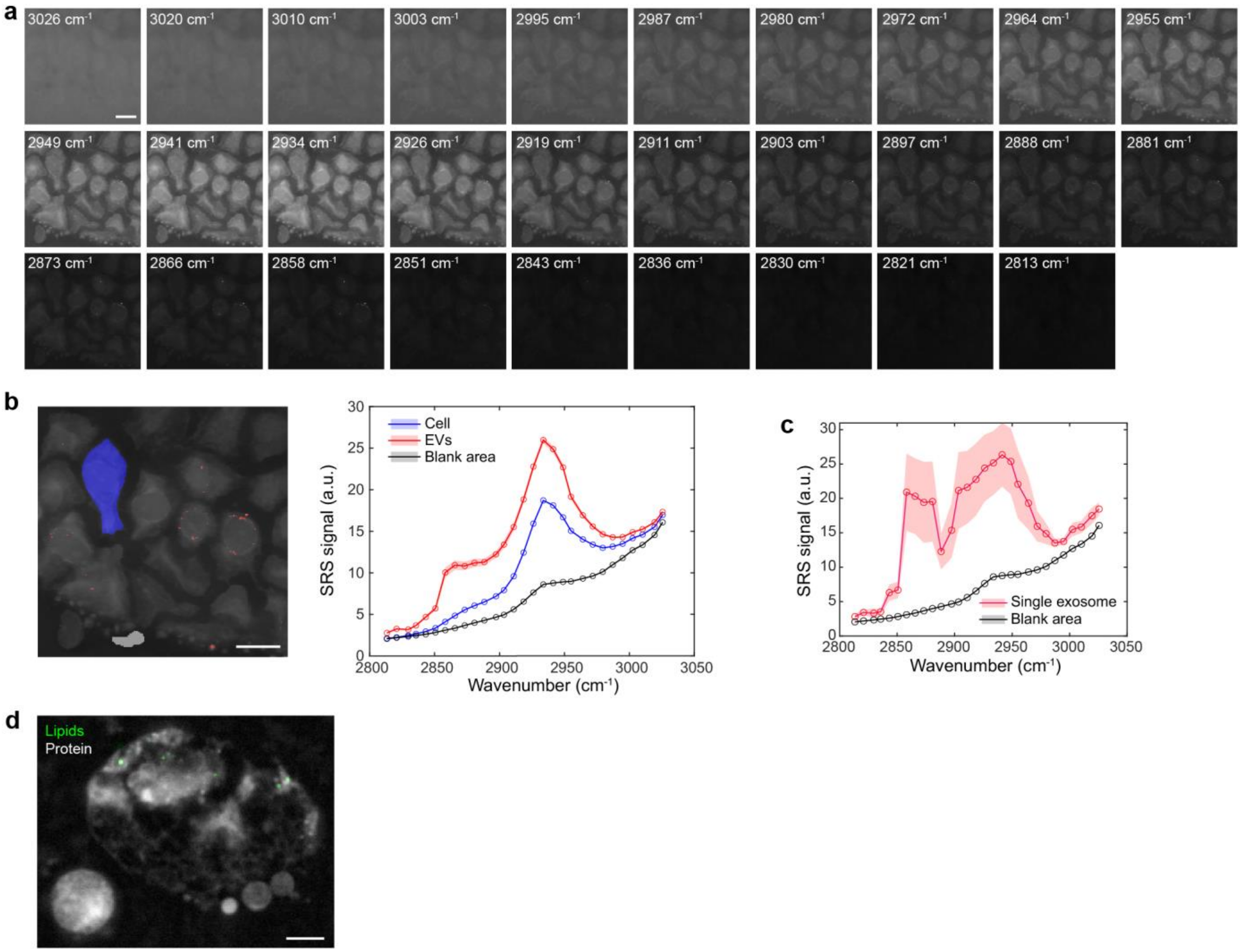

Supplementary Figure 6. Chemical composition study in expanded human bronchial epithelial cells-derived lung organoid. a, Images of hyperspectral SRS acquisition on the human lung organoid. The wavenumbers were labeled at the up-left corner of each image. **b**, Hyperspectral SRS spectra of cell area (blue), extracellular vesicles (EVs, red) and blank gel area (gray) of the data cube in (**a**). Selected areas were labeled on the left image. **c**, Hyperspectral SRS spectra of a single EV marked by a pink arrow in Fig. 3b and blank gel area (gray). Shaded area indicates the s.e.m. of SRS spectrum from different pixels. **d**, Overlay image of lipids and protein channel indicates some small dots in a large exosome are rich in lipids. All images were acquired with a 1.05 NA objective in 1× PBS (expansion factor, 4.5). Scale bars (in biological scales), 10 μm in (**a**, **b**); 1 μm in (**d**).

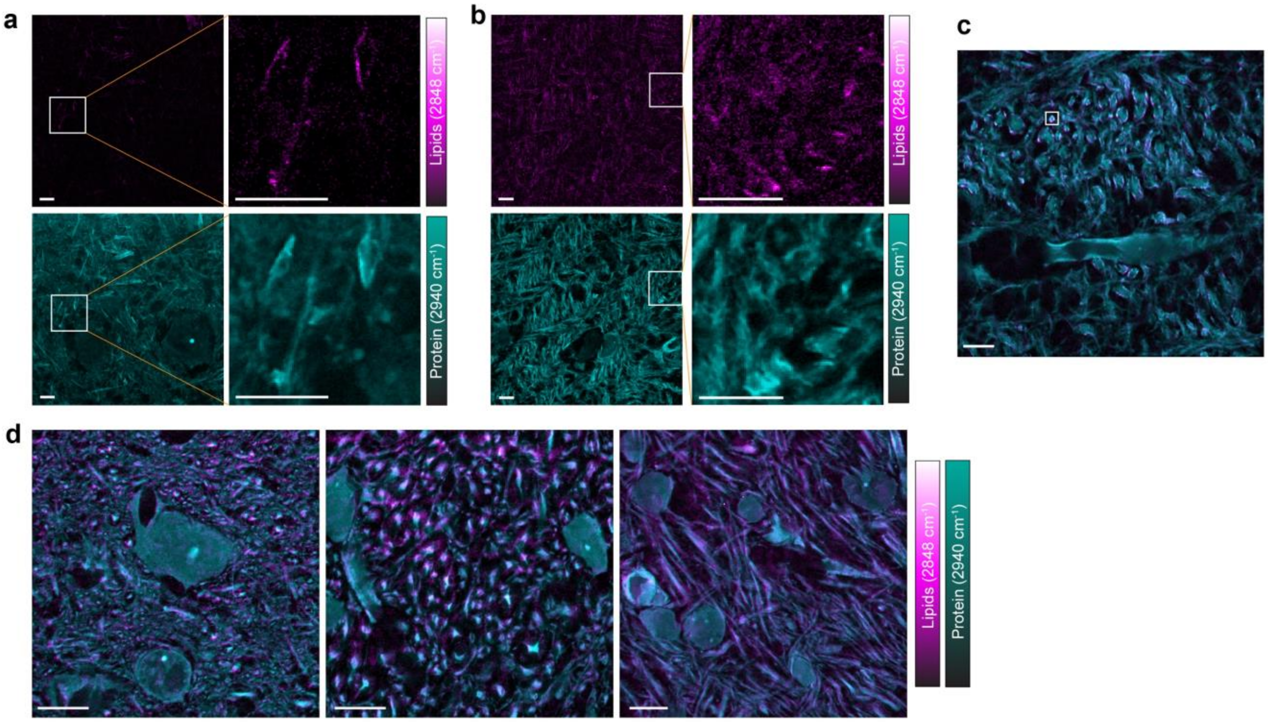

Supplementary Figure 7. Imaging of protein and lipids in the expanded mouse brain tissue. Representative protein and lipid channels in expanded mouse brain (**a**-**c**) 2.56-fold expansion, (**d**) 4.88-fold expansion. The white box in (**c**) is magnified in Fig. 3g. All images were acquired with a 25× objective. Scale bars (in biological scales), 5 μm.

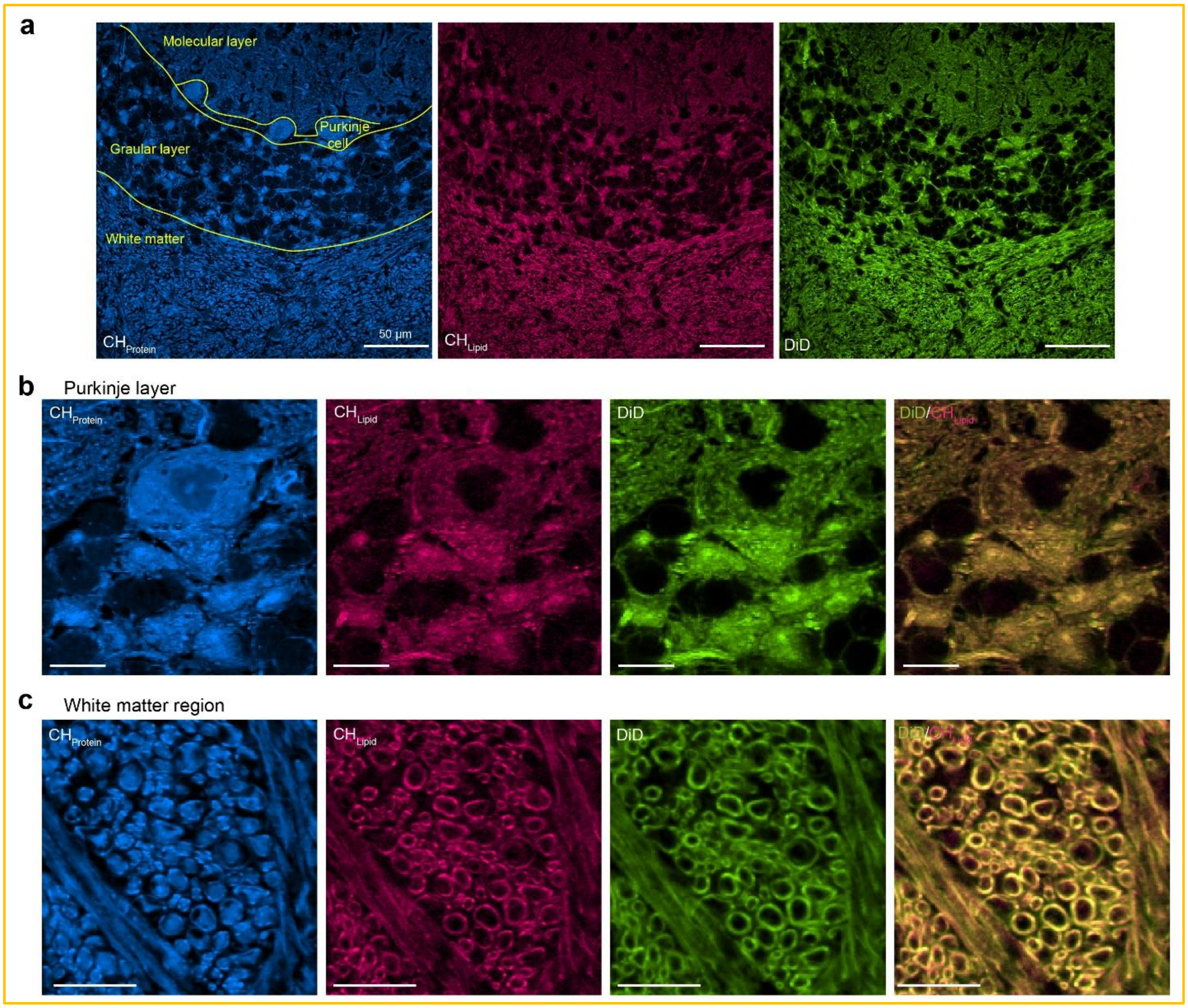

Supplementary Figure 8. DiD staining validation of lipid imaging. $CH_{Protein}$, CH_{Lipid} and DiD fluorescence images of expanded mouse cerebellum. Histological structures are labeled in the $CH_{Protein}$ channel in (**a**). Scale bars (in biological scales), 50 µm in (**a**); 10 µm in (**b-c**).

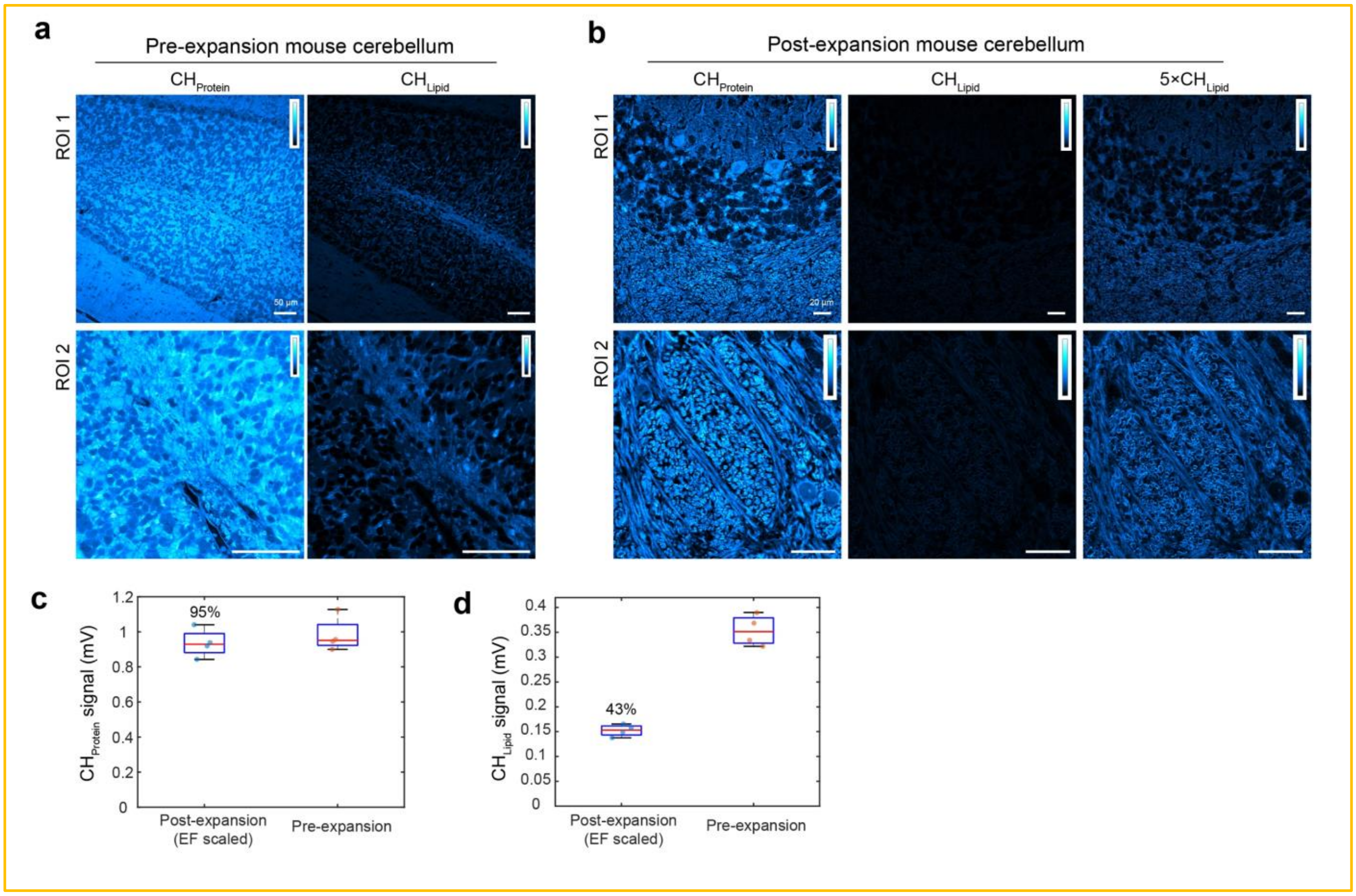

Supplementary Figure 9. Evaluation of lipid retention on mouse cerebellum tissue sections. **a**, $CH_{Protein}$ and CH_{Lipid} images of pre-expansion untreated tissue. **b**, $CH_{Protein}$ and CH_{Lipid} images of post-expansion tissue sample (expansion factor ~1.6). **c**-**d**, Quantification of (**c**) protein and (**d**) lipid retention rate in mouse cerebellum tissues. SRS signals were plotted as mean$\pm$s.d. (n=4, 4 regions of interest (ROIs)). Absolute signal in the expanded sample is converted with a volume dilution factor of 2^3= ~8-fold after expansion. Scale bars (in biological scales), 50 μm in (**a**); 20 μm in (**b**).

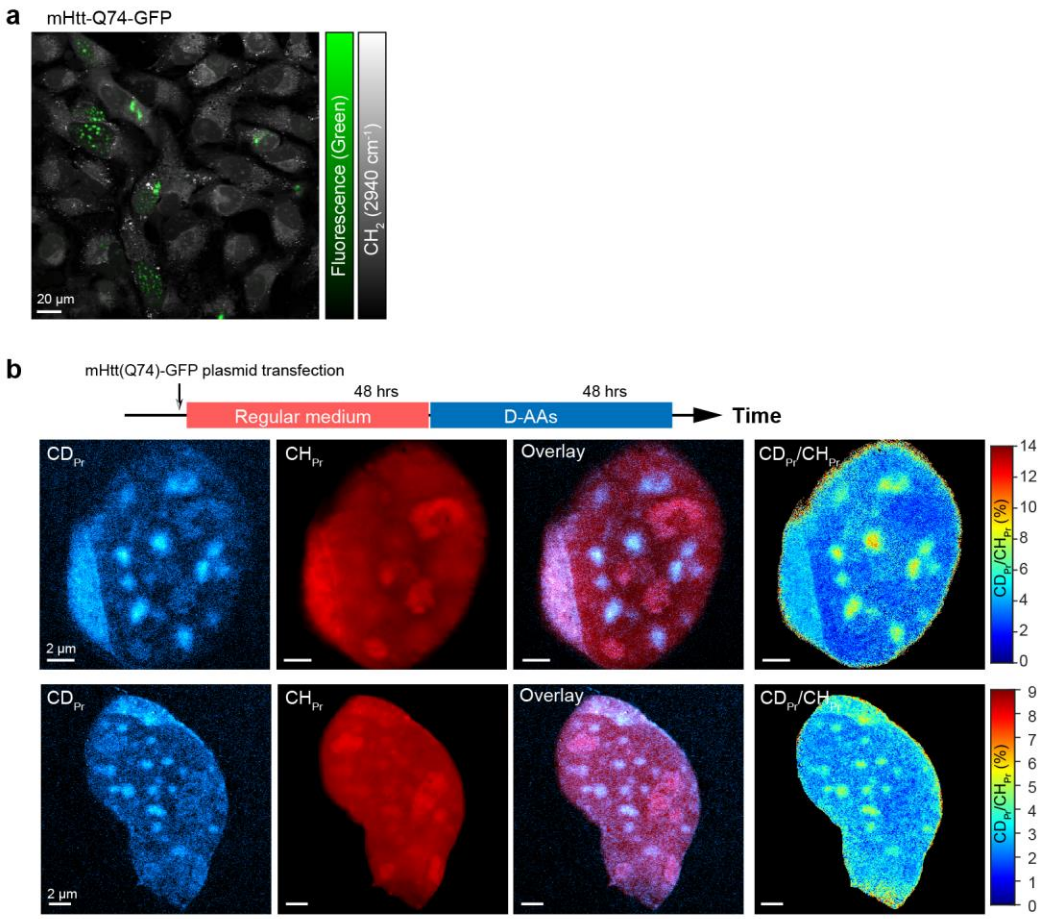

Supplementary Figure 10. Nanoscale imaging of protein metabolism in Huntingtin aggregates with isotope labeling. a, Expression of mutant huntingtin polyQ protein was confirmed with both fluorescence (green channel) and SRS (CH_3 channel, 2941 cm^{-1}) imaging before expansion. Deuterated amino acid labeling in time with simultaneous expression of mutant huntingtin (mHtt74Q-GFP) proteins for 48 hrs. **b**, Regular DEME medium was applied with simultaneous expression of mutant huntingtin (mHtt74Q-GFP) proteins for 48 hrs, chased with deuterated amino acid labeling for another 48 hrs. Images were acquired with a 25× objective in 5× PBS (expansion factor, 3.44). Scale bars (in biological scales), 20 µm in (**a**); 2 µm in (**b**).

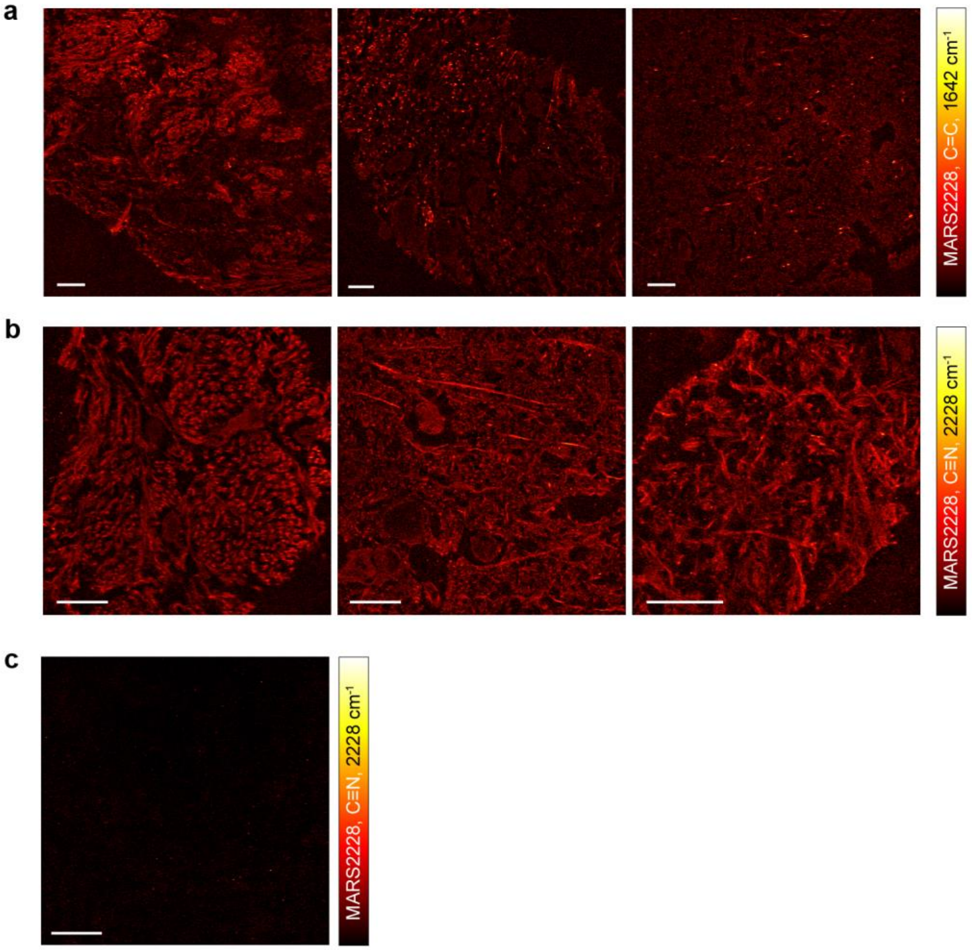

Supplementary Figure 11. Suppression of non-specific staining of MARS dyes. a-b, Nonspecific staining backgrounds were observed when 1× PBS was used as the staining buffer. In (**a**), expanded mouse brain tissue was first labeled with rabbit anti-Synaptophysin followed by labeling with donkey-anti-rabbit MARS2228 antibody. SRS images were acquired by probing the C=C bond at 1642 cm^{-1}. In (**b**), expanded mouse brain tissue was first stained with mouse anti-α tubulin followed by labeling with donkey-anti-mouse MARS2228 antibody. SRS images were acquired by probing a nitrile bond at 2228 cm^{-1}. **c**, Correct staining patterns of Synaptophysin using 9× PBS with 10% Triton-X as the staining buffer. Expanded mouse brain tissues stained with rabbit anti-Synaptophysin primary antibody and donkey-anti-rabbit MARS2228 antibody and imaged via the nitrile bond at 2228 cm^{-1}. Scale bars (in biological scales), 10 µm.

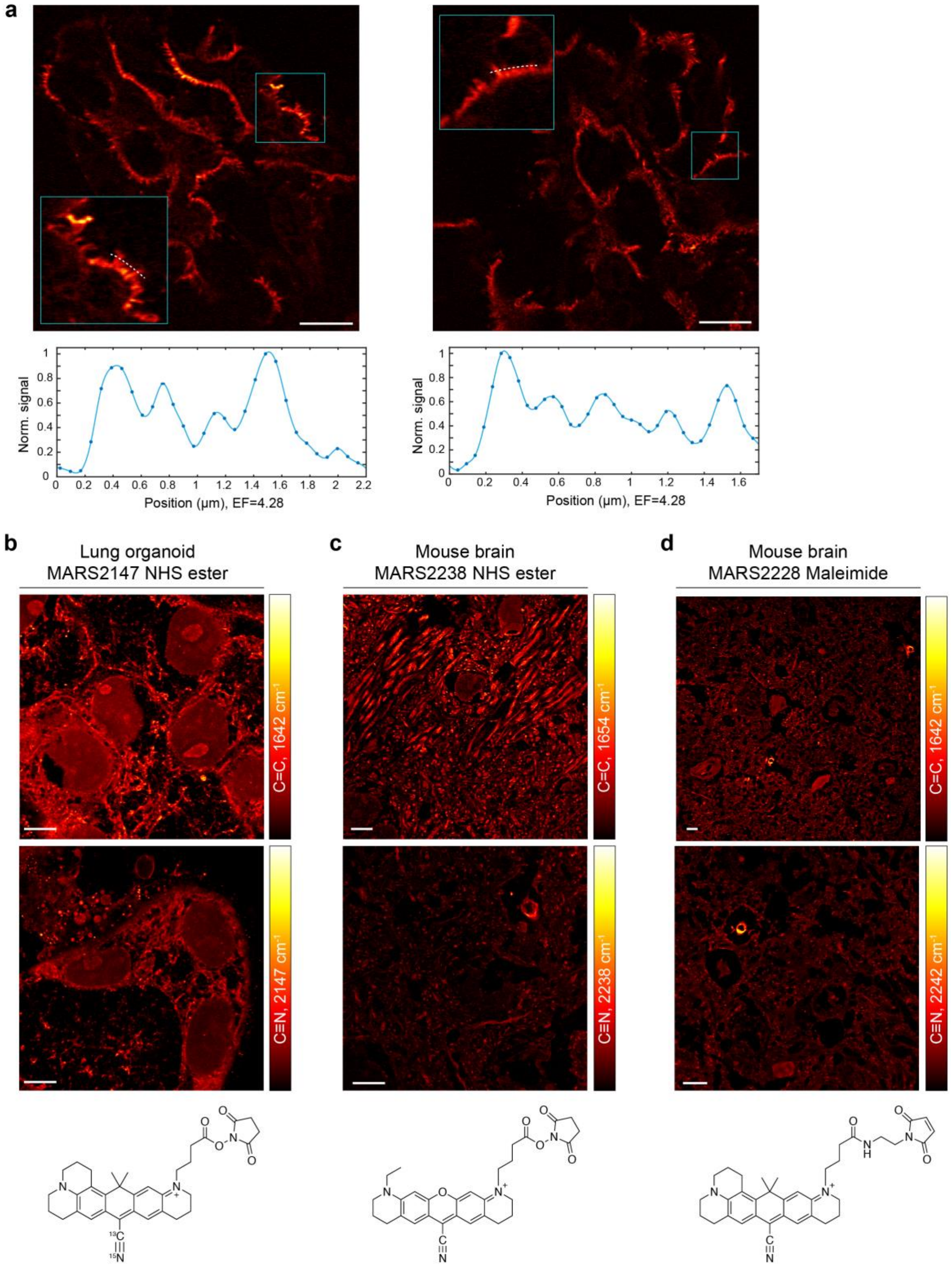

Supplementary Figure 12. Epr-SRS imaging of immuno-staining and pan-staining with MARS probes. a, Immuno-eprSRS of actinin-4 (ACTN4, specifically label tertiary podocyte foot processes) in human kidney FFPE tissue with MARS2228. Inset, zoom into the region outlined by the blue box, dotted white curve within the inset indicates the line cut analyzed below. Below, normalized epr-SRS signal along the line cut of the inset. **b-d**, Pan-staining with (**b**) NHS-ester-functionalized MARS2147 on expanded human lung organoid, (**c**) NHS-ester-functionalized MARS2238 on expanded mouse brain tissue and (**d**) Maleimide-functionalized MARS2228 on expanded mouse brain tissue. Chemical

structures of MARS probes were shown below images. Images were acquired with a 25× objective. (**a**) were imaged in 1/50 PBS (4.28-fold expansion, human kidney ExPath gel); (**b-d**) were imaged in 1× PBS (4.5-fold expansion, mouse brain MAGNIFY gel). Scale bars (in biological scales), 5 μm in (**b-d**).

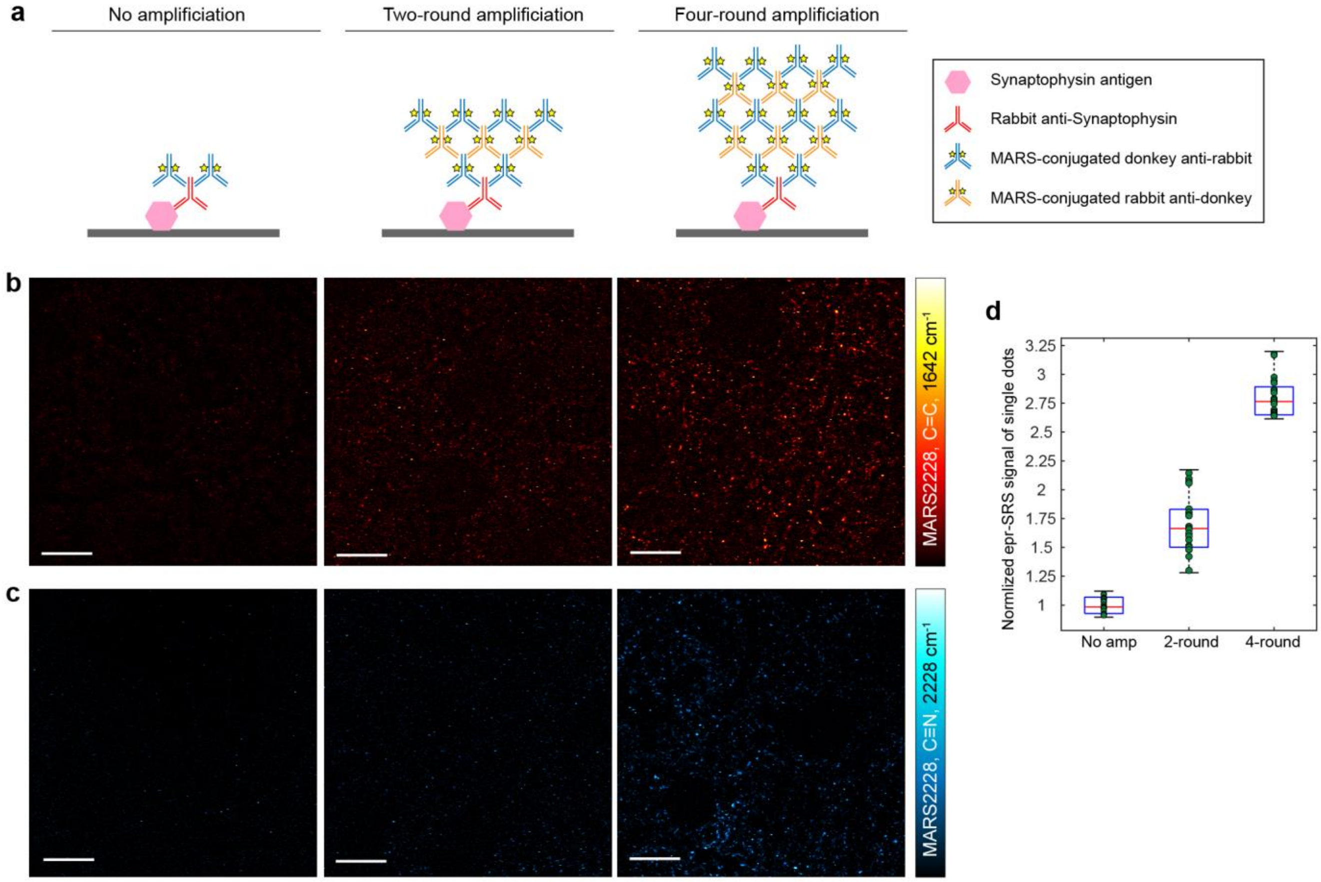

Supplementary Figure 13. Signal amplification via cyclic staining. a, Schematic explanation of signal amplification of epr-SRS via cyclic staining of MARS-conjugated secondary antibodies. **b-c**, Epr-SRS images of Synaptophysin in mouse brain via (**b**) C=C bond at 1642 cm^{-1} and (**c**) C≡N bond at 2228 cm^{-1} of MARS2228. Left to right: no amplification, amplification with additional two rounds staining and amplification with additional four rounds staining. **d**, Quantification of amplification factors (mean±s.d.) with C=C band at 1628 cm^{-1} of MARS2228. Strongest 20 dots were selected for analysis in each condition. Images were acquired with a 25× objective in 1× PBS (4.5-fold expansion). Scale bars (in biological scales), 10 μm.

Supplementary Table 1. Reagents

Name	Source	Catalog No.
A8301 (inhibitor of transforming growth factor β kinase type 1 receptor)	Sigma-Aldrich	SML0788-5MG
BEBM™ Bronchial Epithelial Cell Growth Basal Medium	Lonza	CC-3171
BEGM™ Bronchial Epithelial Cell Growth Medium SingleQuots™ Supplements and Growth Factors	Lonza	CC-4175
Dulbecco's Modified Eagle Medium	Gibco™	11965
CHIR99021 (activator of WNT pathway)	Reprocell	04000402
Dulbecco’s Phosphate-Buffered Saline (DPBS), 1X without calcium and magnesium	Corning	21-031-CV
DMH-1 (Inhibitor of BMP4/SMAD signaling)	Tocris Bioscience	4126
Growth factor reduced Matrigel	Corning	CB 40230
Heparin solution	Stemcell Technologies	07980
HyClone™ FetalClone™ I Serum (a fetal bovine serum alternative)	GE Healthcare	SH30080.03
Hydrocortisone stock solution	Stemcell Technologies	07925
Normal Human Bronchial Epithelial (NHBE) cells without Retinoic Acid	Lonza	CC-2541
Paclitaxel	Cayman Chemicals	10461
PneumaCult™-ALI Basal Medium	Stemcell Technologies	05002
PneumaCult™-ALI 10X Supplement	Stemcell Technologies	05003
PneumaCult™-ALI Maintenance Supplement	Stemcell Technologies	05006
Penicillin-Streptomycin	Lonza Walkersville Inc.	17-602E
RPMI 1640 with L-glutamine	Corning	10-040-CV
Y27632 (Inhibitor of ROCKs)	Cayman Chemical	129830-38-2

0.25% Trypsin-EDTA	Gibco™	25200056
Proteinase K	Thermo Scientific	EO0491
Propyl gallate	Sigma Aldrich	P3130

Supplementary Table 2. Calibrated expansion factors of various samples in different buffer conditions

Sample type	Measured buffer condition	Expansion factor			Calculation method utilized
		Mean	STE	N	
Mouse kidney	1× PBS	4.30	0.07	15	Distances
	1:25× PBS	7.43	0.27	10	
	1:50× PBS	8.10	0.19	12	
Mouse brain (ExPath Gel[1])	1× PBS	1.99	0.24	1724	
	1:25× PBS	2.56	0.37	1062	
HeLa cells	5× PBS	3.44	0.68	110	
Mouse brain (MAGNIFY Gel[2])	10× PBS	2.96	0.68	82	
	5× PBS	3.23	0.63	189	
	2× PBS	3.85	0.56	414	
	1× PBS	4.48	0.62	403	
	1:10× PBS	5.38	0.82	163	
	1:50× PBS	7.18	1.22	165	
	Water	10.13	1.60	133	

[1]ExPath Gel formula: 15 g/100mL Sodium Acrylate, 5 g/100 mL Acrylamide, 500 ppm N,N′-Methylenebisacrylamide (Bis), 11.7 g/100 mL NaCl, 1x PBS.
[2]MAGNIFY Gel Formula: 4 g/100 mL N,N-Dimethylacrylamide (DMAA), 34 g/100 mL Sodium Acrylate, 10 g/100 mL Acrylamide, 100 ppm N,N′-Methylenebisacrylamide (Bis), 1 g/100 mL NaCl, 1x PBS.

Supplementary Table 3. Antibody summary

**Primary antibodies**					
Target	Vendor	Catalog #	Host species[a]	Concentration (mg/mL)	Clonality[b]
ACTN4	Sigma	HPA001873	Rabbit	0.2	pAb
MAP2	SYSY	188004	Guinea Pig	/	pAb
Synaptophysin	Proteintech	17785-1-AP	Rabbit	/	pAb
PSD95	Abcam	ab12093	Goat	1	pAb
TH	Abcam	ab76442	Chicken	0.2	pAb
αTubulin	Sigma	T6199	Mouse	1	mAb
Vimentin	Abcam	ab24525	Chicken	/	pAb

**Secondary antibodies**		
Target	Source	Catalog #
Bovine anti-Goat IgG (H+L)	Jackson ImmunoResearch	805-005-180
Rat anti-Mouse IgG (H+L)	Jackson ImmunoResearch	415-005-166
Goat anti-Bovine IgG (H+L)	Jackson ImmunoResearch	101-005-165
Mouse anti-Rat IgG (H+L)	Jackson ImmunoResearch	212-005-168
Donkey anti-Rabbit IgG (H+L)	Jackson ImmunoResearch	711-005-152
Rabbit anti-Donkey IgG (H+L)	Thermo Fisher	SA1-26816
Donkey anti-Guinea Pig IgG (H+L) Alexa Fluro 488	Jackson ImmunoResearch	706-545-148
Donkey anti-Chicken IgY (H+L) Cy3	Jackson ImmunoResearch	703-165-155
Goat anti-Rabbit IgG (H+L)	Thermo Fisher	SA5-10041

**Lectins**				
Target	Conjugate	Source	Catalog #	Concentration (mg/mL)
Lycopersicon Esculentum lectin	DyLight488	Vector	DL-1174	1
Lycopersicon Esculentum lectin	unconjugated	Vector	L-1170	/
Wheat Germ Agglutinin	unconjugated	Sigma Aldrich	L0636	/

**Non-protein based**		
Name	Source	Catalog #
NucBlue Fixed Cell	Invitrogen	R37606
DAPI	Thermo Fisher	62248

Supplementary Note

Possible mechanisms of lipid and DNA anchoring with methacrolein.

MAGNIFIERS uses MAGNIFY expansion protocol[5] where a small molecule used in classic fixation protocols called methacrolein (structure in Fig.1) in the monomer solution to incorporate more biomolecules to the gel. There are several possible mechanisms that lead to post-expansion lipids and DNA retention. The monomer methacrolein is a fixative that is more reactive than widely used paraformaldehyde[6-8]. Primary amines in the head group of lipids (such as phosphatidylserine (PS) and phosphatidylethanolamine (PE)) and nucleic acid bases (like adenine, guanine, cytosine) can react with the aldehyde group in methacrolein in the monomer solution and crosslink to proteins or the gel polymer chains. Moreover, methacrolein can generate radicals that crosslink the C=C bond to the gel. On the other hand, some lipids and nucleic acids that bind to proteins (like lipoproteins and DNA-protein complex) may be retained in the gel as the interacting proteins are anchored. Notably, MAGNIFY retains near 100% proteins (Fig. 1c-d) which could be promising to give better lipids and DNA retention. Certainly, a portion of unreacted lipids will remain mobile and hence be solubilized and washed out of the gel upon homogenization and expansion. Based on the SRS measurement, we found ~43% lipids are maintained inside the gel after expansion for mouse brain tissues (Supplementary Fig. 9d).

Supplementary Video 1. 3D-rendered SRS images of CH_3 peak at 2941 cm^{-1} of an expanded mouse brain tissue (2-fold expansion) taken at 25× magnification.

Supplementary Video 2. 3D-rendered SRS images of CH_3 peak at 2941 cm^{-1} of an expanded human lung organoid (4.5-fold expansion) taken at 25× magnification.

Supplementary Video 3. 3D-rendered epr-SRS image of MARS2228-conjugated *Lycopersicon Esculentum* lectin (LEL) labeling (red) and CH_3 image of 2941 cm^{-1} (green) of an expanded mouse brain tissue (4.5-fold expansion) taken at 25× magnification.

Supplementary Video 4. 3D-rendered epr-SRS image of expanded human lung organoid (4.5-fold expansion) stained with MARS2147 NHS ester dye taken at 25× magnification.

Supplementary Video 5. 3D-rendered epr-SRS image of MARS probe immunolabeled Synaptophysin (green) and PSD95 (red) of an expanded mouse brain tissue (4.5-fold expansion) taken at 25× magnification.

Supplementary References

1. Miao Y, Qian N, Shi L, Hu F, Min W. 9-Cyanopyronin probe palette for super-multiplexed vibrational imaging. *Nat Commun* **12**, 4518 (2021).
2. Levardon H, Yonker LM, Hurley BP, Mou H. Expansion of airway basal cells and generation of polarized epithelium. *Bio-protocol* **8**, e2877 (2018).
3. Wei L, *et al.* Imaging complex protein metabolism in live organisms by stimulated Raman scattering microscopy with isotope labeling. *ACS Chem Biol* **10**, 901-908 (2015).
4. Wei L, *et al.* Super-multiplex vibrational imaging. *Nature* **544**, 465-470 (2017).
5. Klimas A, *et al.* Nanoscale imaging of biomolecules using molecule anchorable gel-enabled nanoscale in-situ fluorescence microscopy. *Nat Portfolio*, 10.21203/rs.3.rs-858006/v1, (2021).
6. Sabatini DD, Bensch K, Barrnett RJ. Cytochemistry and electron microscopy: The preservation of cellular ultrastructure and enzymatic activity by aldehyde fixation. *J Cell Biol* **17**, 19-58 (1963).
7. Jones D. Acrolein as a histological fixative. *Journal of Microscopy* **90**, 75-77 (1969).
8. Saito T, Keino H. Acrolein as a fixative for enzyme cytochemistry. *Journal of Histochemistry & Cytochemistry* **24**, 1258-1269 (1976).
